# A digital pathology tool for quantification of color features in histologic specimens

**DOI:** 10.1002/btm2.10242

**Published:** 2021-08-24

**Authors:** Melanie Reschke, Jenna R. DiRito, David Stern, Wesley Day, Natalie Plebanek, Matthew Harris, Sarah A. Hosgood, Michael L. Nicholson, Danielle J. Haakinson, Xuchen Zhang, Wajahat Z. Mehal, Xinshou Ouyang, Jordan S. Pober, W. Mark Saltzman, Gregory T. Tietjen

**Affiliations:** ^1^ Department of Molecular Biophysics & Biochemistry Yale University New Haven Connecticut USA; ^2^ Department of Surgery Yale School of Medicine New Haven Connecticut USA; ^3^ Department of Biomedical Engineering Yale University New Haven Connecticut USA; ^4^ Department of Surgery University of Cambridge Cambridge UK; ^5^ Department of Pathology Yale School of Medicine New Haven Connecticut USA; ^6^ Section of Digestive Diseases, Department of Internal Medicine Yale University School of Medicine New Haven Connecticut USA; ^7^ Department of Immunobiology Yale University New Haven Connecticut USA

**Keywords:** color image analysis, histology, human organ research, immunohistochemistry

## Abstract

In preclinical research, histological analysis of tissue samples is often limited to qualitative or semiquantitative scoring assessments. The reliability of this analysis can be impaired by the subjectivity of these approaches, even when read by experienced pathologists. Furthermore, the laborious nature of manual image assessments often leads to the analysis being restricted to a relatively small number of images that may not accurately represent the whole sample. Thus, there is a clear need for automated image analysis tools that can provide robust and rapid quantification of histologic samples from paraffin‐embedded or cryopreserved tissues. To address this need, we have developed a color image analysis algorithm (DigiPath) to quantify distinct color features in histologic sections. We demonstrate the utility of this tool across multiple types of tissue samples and pathologic features, and compare results from our program to other quantitative approaches such as color thresholding and hand tracing. We believe this tool will enable more thorough and reliable characterization of histological samples to facilitate better rigor and reproducibility in tissue‐based analyses.

**Abbreviations**
DigiPath: digital pathology tool
DBD: donation after brain death
DCD: donation after cardiac death
CSF: cerebrospinal fluid
CVA: cerebrovascular
FFPE: formalin‐fixed, paraffin‐embedded
H&E: hematoxylin and eosin
HFD: high fat diet
HTK: histidine‐tryptophan‐ketoglutarate
J‐score: Youden's J statistic
J‐score: Youden's J statistic
LFT: liver function test
MSB: martius scarlet blue
NASH: non‐alcoholic steatohepatitis
NEDS: New England Donor Services
RGB: red/green/blue
TUNEL: terminal deoxynucleotidyl transferase dUTP nick end labeling

## 1 INTRODUCTION

Histological analysis of tissue biopsies—either formalin‐fixed, paraffin‐embedded (FFPE), or cryopreserved—remains a cornerstone of preclinical research.^1^, ^2^, ^3^, ^4^, ^5^ Nevertheless, there are well acknowledged issues with the reliability of the standard semiquantitative assessments typically performed on these samples.^2^, ^6^, ^7^ These issues can potentially be circumvented in clinical settings by carefully controlled workflows that ensure consistency in sample preparation and reduce interobserver variability.^8^ However, replicating these conditions in preclinical research often presents a variety of challenges depending on the nature of the analysis to be performed and whether the researchers have access to an expert pathologist.^4^

Even if researchers have access to a trained pathologist, the volume of tissue typically involved in preclinical research can make manual analysis of each individual image impractical. Studies in animal models with large numbers of replicates can yield an overwhelming abundance of tissue. Similarly, studies utilizing non‐transplanted human organs—an area of emphasis in our laboratory—require evaluation of large amounts of tissue per organ in order to properly characterize these highly heterogenous samples. In two recent examples, we evaluated 1000's of images collected from dozens of biopsies.^9^, ^10^ In these instances, manual forms of color image analysis—where the researcher must evaluate each individual image one by one—are simply not feasible.

Digital color thresholding is a commonly used method to automate the analysis of features of interest in color images of histologic samples.^4^, ^11^, ^12^, ^13^ In these methods, a threshold value is identified for each of the three RGB (red, green, and blue) color channels to isolate a specific subset of color shades. However, color features in standard histologic stains (e.g., hematoxylin and eosin [H&E]) are typically blended shades of the three RGB colors. As a result, what is visually distinct to the eye can be difficult or even impossible to isolate using a simple color thresholding approach. To overcome this limitation, more sophisticated approaches have been developed that can identify single features of interest within specific stains.^14^, ^15^, ^16^ While useful for certain focused applications, these approaches are limited by their lack of adaptability to any color feature of interest regardless of histochemical stain. Thus, there remains a need for software that has the efficiency and reproducibility of the existing automated methods but is also easily adaptable to many different types of histological features/stains within a single workflow.

The aim of this work was to address the need for more rapid, reliable, and adaptable methods of digital analysis in histological specimens. To that end, we developed and validated an automated image analysis approach (DigiPath) that uses a color‐based classification algorithm to identify and rapidly quantify areas of interest in color images. We demonstrate that this approach is accurate, reliable, and significantly faster than a standard method of hand tracing areas of interest. We also show that it can be used for assessment of a wide array of different histological features in human and animal biopsy specimens. Based on the evidence presented here, we believe DigiPath can enable comprehensive, reproducible, and rapid analysis of histology specimens in preclinical research.

## 2 RESULTS

### 2.1 DigiPath yields more efficient results when compared to hand tracing methods

**FIGURE 1:**
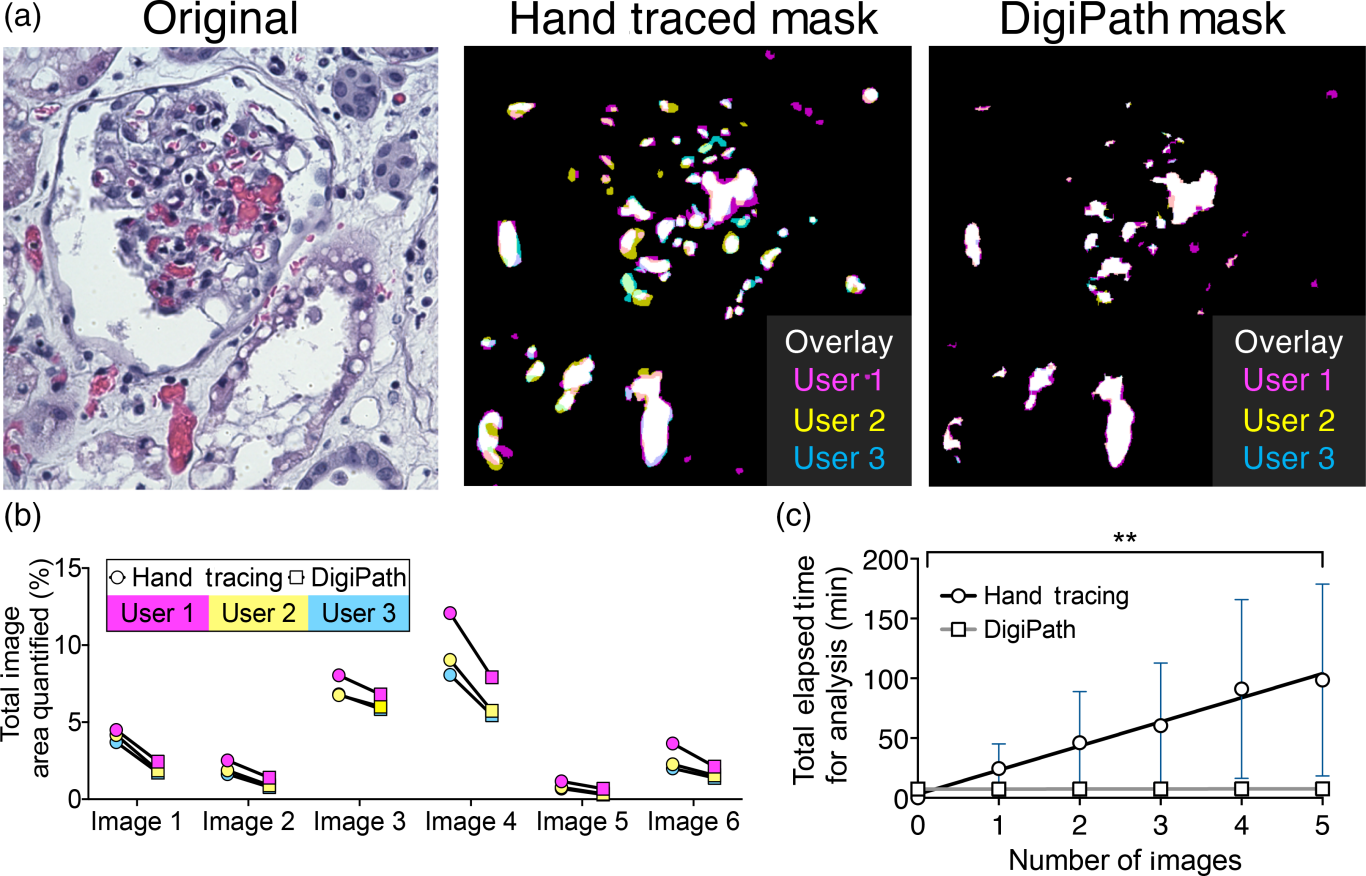
DigiPath is a more efficient method for quantification than hand tracing. (a) Representative image from an H&E section of a kidney during normothermic machine perfusion (NMP). Obstructions are quantified using hand tracing or DigiPath by three individual users (User 1—magenta, User 2—yellow, User 3—cyan). (b) Total area quantified using hand tracing or DigiPath methods from three users. (c) Total time elapsed for hand tracing (circles, black line) or DigiPath (squares, gray line) methods across three users for five separate images. Mixed‐model ANOVA showed a significant difference between the DigiPath and hand tracing cumulative analysis times (**p = 0.0027)

Hand tracing is frequently used as a standard method to quantify areas of interest in IHC stained tissue sections from preclinical biopsy specimens. We therefore compared DigiPath to traditional hand tracing as a gold standard. Three independent users were asked to quantify areas of microvascular obstruction in human kidney biopsies that underwent normothermic machine perfusion (NMP) using both hand tracing in ImageJ and DigiPath. These microvascular obstructions (not classic thrombi) are structures unique to the serum free perfusate conditions of NMP and are easily identifiable on histology.^9^ With DigiPath, we observed that features were systematically ~1%–3% smaller as compared to hand tracing. This result is consistent with a small and systematic over estimation of size by users when hand tracing features (Figure 1a and Supplemental Figure 1). We also found that images with more positive area (e.g., Image 4) were subjected to higher inter‐user variability (~21% error of the mean) with either method (Figure 1b). Nevertheless, there was general agreement in quantified area between hand tracing and DigiPath methods (Figure 1b). Although inter‐user variability was observed, we found that each user reported similar relative trends in the amount of positive area (i.e., User 1 values > User 2 values > User 3 values).

While DigiPath produced similar results to hand tracing, we found that DigiPath significantly reduced the time of analysis. To quantify five images by hand, users took on average 98.5 ± 80.3 min. However, when using DigiPath to quantify those same five images, users took on average 7.6 ± 1.7 min in total. This number includes the time it took users to set parameters in training images and process images of interest. A repeated measure mixed‐model two‐way analysis of variance (ANOVA) showed that the method of analysis (i.e., DigiPath vs. hand tracing) significantly affected cumulative analysis time (Figure 1c). We also conducted a Bonferroni multiple comparisons test to evaluate the difference in cumulative analysis times between DigiPath and hand tracing at each image number up to five images. Due to the high variability between users in the hand tracing analysis time, at five images the difference in cumulative analysis time between DigiPath and hand tracing was not statistically significant (*p* = 0.062). However, we observed a trend of decreasing *p* values between DigiPath and hand tracing as the number of images increased (three images: *p* = 0.613, four images: *p* = 0.098, five images: *p* = 0.062).

We next extrapolated how long it might take to analyze 500 images, a typical number of images in our prior studies. We estimate that we would save at least ~167 h of quantification time compared to hand tracing (Supplemental Figure 2). Analyzing 500 images by hand tracing is unrealistic and would be unlikely to be carried out in a study. Nonetheless the extrapolation from the average hand tracing time per image provides a conservative estimate of the amount of time that can be saved by quantifying color image features with DigiPath. It also demonstrates how DigiPath analysis can enable a far more in‐depth quantitative analysis than is practical with a manual approach.

### 2.2 DigiPath achieves greater correlation with hand‐traced standards than color thresholding

Standard thresholding methods—which pick a specific threshold value of intensity for each of the three colors to distinguish between feature versus background—are reliable only under conditions where the pathologic features are predominantly a single distinct color (red, green, or blue). However, typical pathologic features (and tissue backgrounds) are mixes of the individual red, green, and blue color channels meaning that they cannot be easily separated this way without either setting the threshold too high (leading to high false negative rates) or setting the threshold too low (leading to high false positive rates). To compare the accuracy of DigiPath to a standard color thresholding approach, three independent users quantified a set of six images using both DigiPath and color thresholding in ImageJ.

**FIGURE 2:**
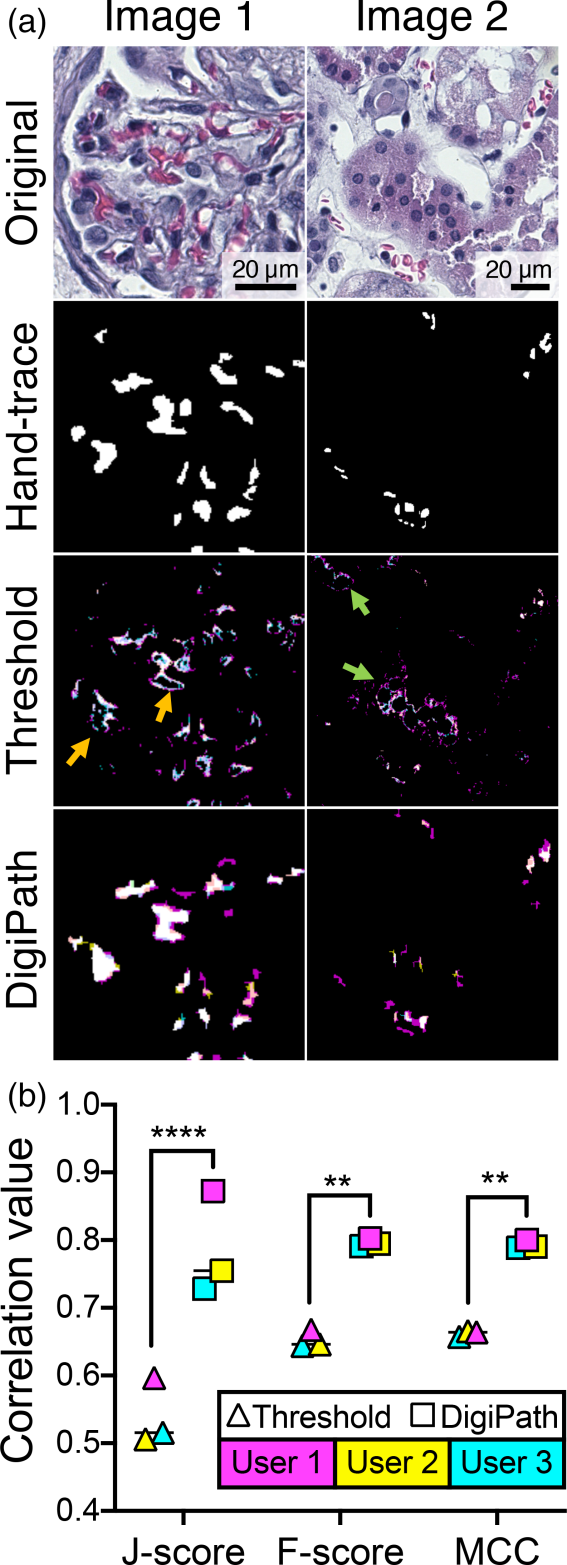
DigiPath achieves better correlation with hand‐traced standards than color thresholding. (a) Representative images of a human kidney section stained with H&E. Masks of microvascular obstructions were generated by hand tracing (a composite of three independent user tracings), color thresholding and DigiPath (overlays of three independent users: User 1—cyan, User 2—yellow, User 3—magenta). Areas of undercounting (orange arrows) and overcounting (green arrows) from color thresholding are shown. (b) F‐score, Matthews correlation coefficient (MCC), and Youden's J statistic were calculated to measure the correlation of results from thresholding and DigiPath methods with the hand‐traced standard. Lines represent median. **p < 0.01; ****p < 0.0001. Scale bars = 20 μm

The results from both DigiPath and standard thresholding were analyzed against a hand‐traced standard. We found that the color thresholding method resulted in a tradeoff between sensitivity (accurate inclusion of all positive regions) and specificity (accurate exclusion of all negative regions). This effect can be seen in the image masks generated from the thresholding results; at the same color threshold setting, some microvascular obstructions are undercounted whereas other areas with no obstruction are incorrectly counted (Figure 2a). Conversely, DigiPath improved sensitivity in identification of positive areas without significantly impacting the exclusion of negative areas.

DigiPath showed greater overall correlation to hand‐traced standards than color thresholding by three metrics: Youden's J statistic (J‐score), F‐score, and Matthew's correlation coefficient (MCC). Each of these metrics are derived from a matrix of all possible classification outcomes (false positives, false negatives, true positives, and true negatives) and are commonly used to analyze the performance of binary classifications.^17^ Across three users, DigiPath consistently classified regions of microvascular obstruction in kidneys with significantly greater correlation to hand‐traced standards than was achieved using color thresholding (Figure 2b).

### 2.3 DigiPath enables quantification of multiple histological features across different stains

**FIGURE 3:**
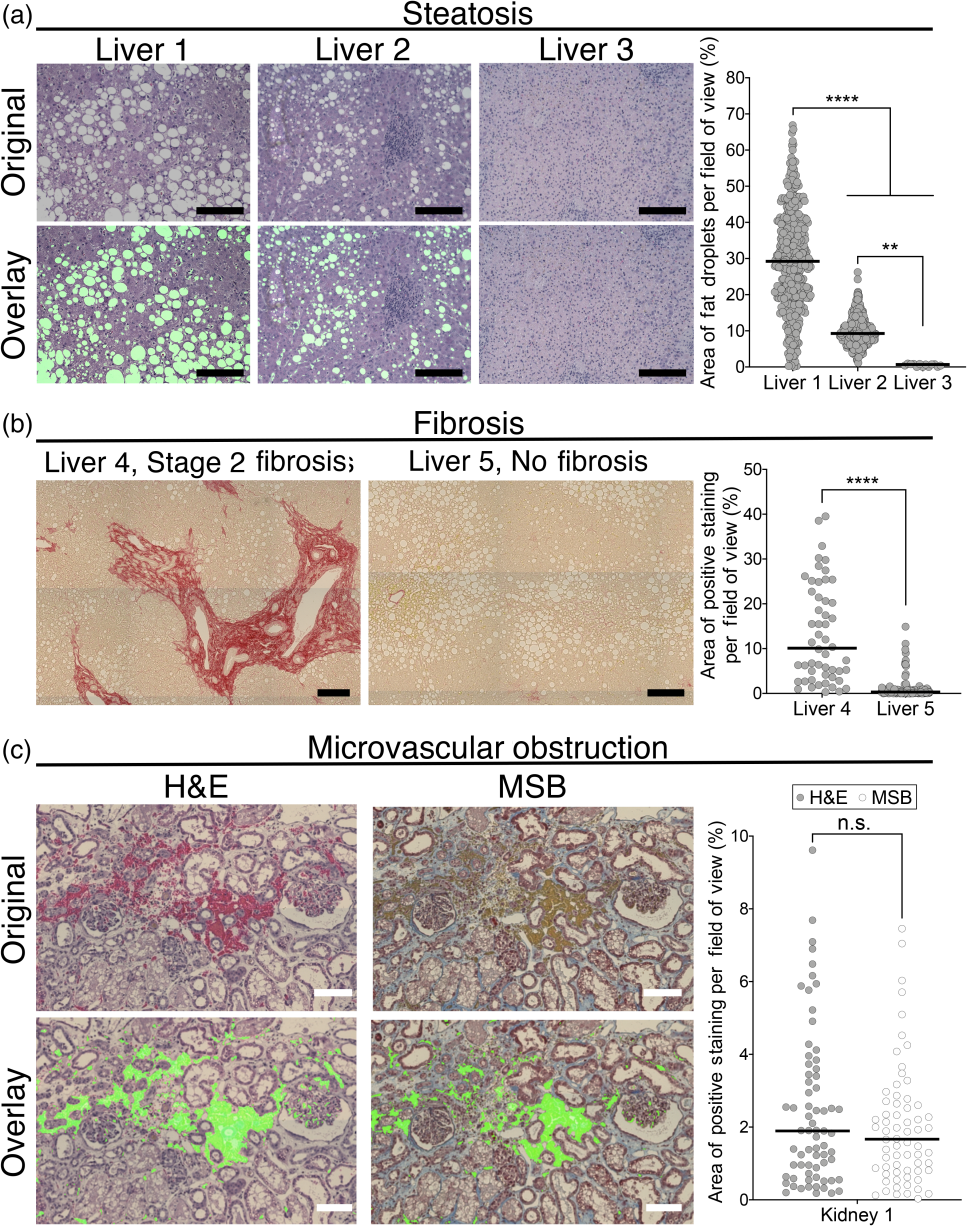
DigiPath enables quantification of multiple histological features across different stains. (a) Representative 20× image fields of three livers with varying degrees of steatosis. Scale bars = 200 μm. Quantification by DigiPath of steatotic area per image field on the right. (b) Representative images of tiled liver biopsies stained with Sirius red. Scale bars = 200 μm. Quantification of Sirius red staining displayed on the right. (c) Representative images of perfused kidneys stained with either H&E (left) or martius scarlet blue (MSB) (right). Scale bars = 100 μm. Overlays show area quantified in a single image. Distribution of positive staining quantified with DigiPath is shown to the right. Each dot represents one field of view. Lines represent the median. Differences between groups are not significant (n.s.) according to a Mann–Whitney test

We next sought to assess the adaptability of DigiPath for quantification of a variety of different histological features between liver and kidney. We first assessed the ability of DigiPath to quantify the degree of steatosis in a series of three transplant‐declined human livers (Figure 3a). Livers 1 and 2 were declined for transplant due to the presence of steatosis, whereas Liver 3 did not list steatosis as a reason for decline (Table 1). We used DigiPath to quantify the area of fat droplets in biopsies from each liver. DigiPath identified fat droplets in both Livers 1 and 2, and negligible droplet area in Liver 3 (Liver 1 median: 29.2%; Liver 2 median: 9.3%; Liver 3 median: 0.7%). Steatosis is reported as the cumulative area of fat droplets per image area (Figure 3a). DigiPath also allows quantification of the variability of steatotic areas within a single biopsy. We used DigiPath to analyze over 400 20× images covering two sections of a biopsy from Liver 1 and found that the percent steatotic area in individual image fields ranged from less than 1% to over 60%, with a median of 29% steatosis (Figure 3a). This demonstrates the capability of DigiPath to characterize the spatial variation of histologic features within a whole biopsy.

**TABLE 1:** Human liver demographics

	Age	Donor type	Cause of death	Reason for decline
Liver 1	50	DBD	CVA	Macrovesicular steatosis ~50% with evidence of NASH
Liver 2	57	DCD	Arrest from presumed electrolyte abnormalities with pancreatitis	Older DCD with alcohol history, steatosis on imaging and abnormal LFTs (peak bili 1.9)
Liver 3	36	DCD	Anoxic arrest (respiratory arrest 2/2 secretions → cardiac arrest)	Slow to progress (extubated 8:55, arrest 9:39, flush 10:13)
Liver 4	49	DBD	CVA witnessed at work, progressed to brain death over days with aggressive care	40%–45% macrovesicular steatosis, moderate inflammation, and stage 2 fibrosis
Liver 5	29	DCD	Known brain aneurysm undergoing elective completion stent assisted coiling with intra‐op aneurysm rupture early March. Complex course with acinetobacter in CSF, vasospasm, and arrhythmias	Transaminases sharply rising in 2–5 k range on 3/14, peaked and coming down

Similarly, we found that we could quantify the distribution of fibrosis in livers using the DigiPath tool on Sirius red stained biopsies. With Sirius red, high collagen levels, associated with fibrosis, stain red in fibrotic and cirrhotic samples. We assessed two livers with stage 2 fibrosis (Liver 4 median: 10.1%) or no diagnosis of fibrosis (Liver 5 median: 0.3%) (Figure 3b) for levels of Sirius red staining. Donor demographics are displayed in Table 1.

We next assessed how reliably DigiPath could quantify the same feature identified with two different stains. To test this, we quantified microvascular obstructions in a perfused kidney using both H&E and MSB stains prepared on sequential sections (Table 2, Figure 3c). Serial sections of a kidney biopsy were stained with either H&E or MSB in order to compare the results from both stains on nearly identical tissue sections. Microvascular obstructions appeared to have similar distributions between the two stains (Figure 3c). According to a Mann–Whitney test, there were no statistical significances between H&E and MSB stained sections. Slight differences in median values may be attributed to the variance of features observed in serial sections and the location of individual fields captured when tiling whole sections.

**TABLE 2:** Human kidney demographics

	Age	Donor type	Cause of death	Reason for decline
Kidney 1	39	DBD	Overdose	Suspected malignancy
Kidney 2	70	DBD	History of hypertension and diabetes	Stroke
Kidney 3	70	DBD	History of hypertension and diabetes	Stroke

### 2.4 DigiPath quantifies steatosis in experimental mouse livers

**FIGURE 4:**
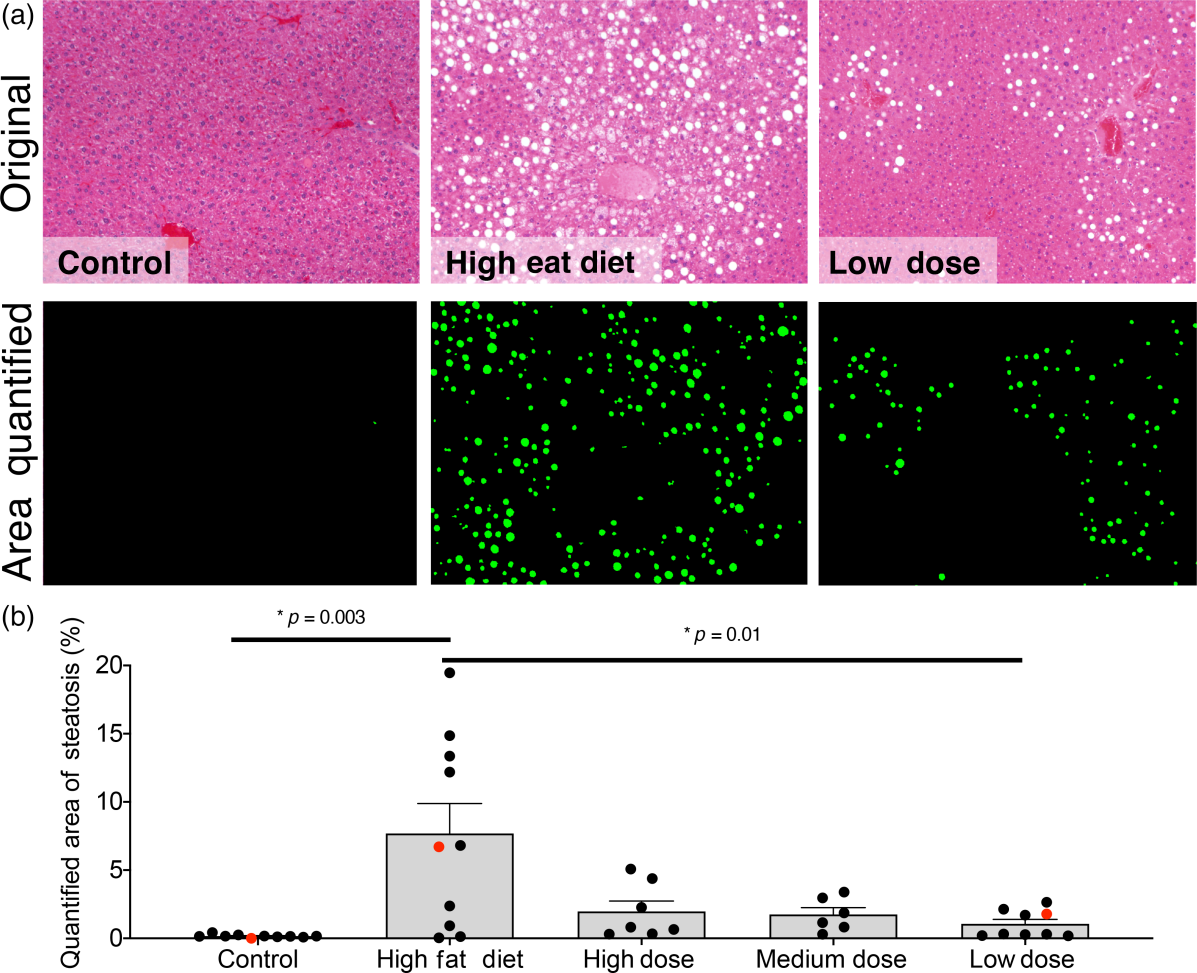
DigiPath quantifies experimental model of mouse hepatosteatosis. (a) Representative images of tissue from mouse livers on a standard diet (control; left), high fat diet (middle), or high fat diet with a low dose of oral Digoxin (right). Area quantified with DigiPath is shown in green. (b) Quantification of steatotic area in murine models of hepatosteatosis with variable doses of Digoxin. Control group was fed standard chow. Each dot represents an individual image. Red dots correspond to images in (a). Error bars represent the standard error of the mean. Differences between groups are significant according to a Student's *t*‐test

To confirm that DigiPath could quantify features of interest from histological specimens processed outside of our lab, we next sought to determine if DigiPath could accurately quantify previously published results.^18^ In a model of murine hepatosteatosis, DigiPath was able to quantify the area of steatosis across a series of images from different animals (Figure 4a,b). DigiPath quantification also confirmed the previously published result that treatment with oral Digoxin reduces hepatosteatosis in mice (Figure 4b).^18^ These results demonstrate DigiPath's utility to quickly and accurately quantify areas of interest across treatment groups in preclinical research models. Additionally, DigiPath's ability to quantify specimens in different species and with variable sample preparations further demonstrates the value of this tool in the preclinical research setting.

### 2.5 DigiPath reveals patterns of cell death in kidney biopsies during cold storage

**FIGURE 5:**
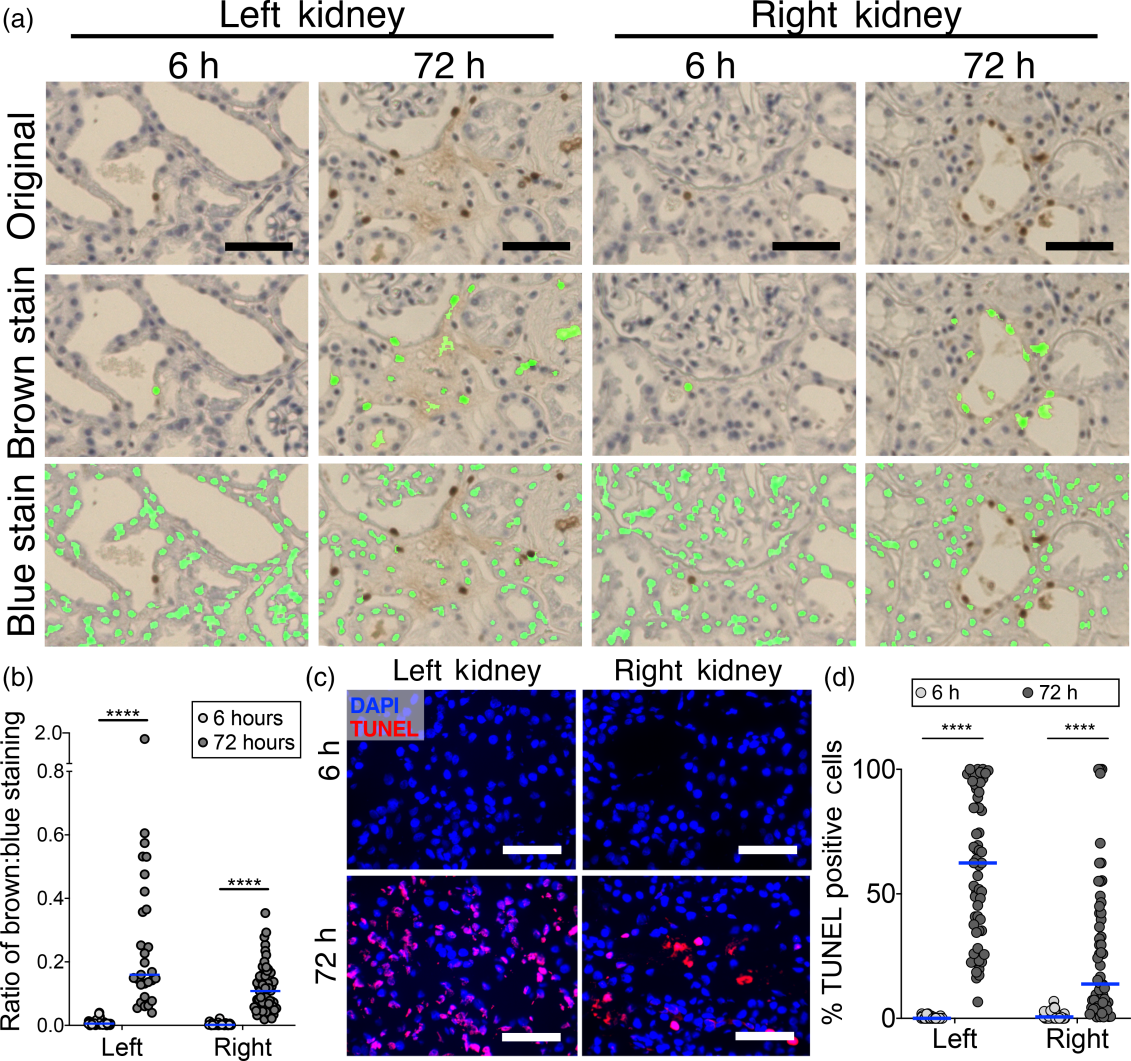
DigiPath reveals patterns of cell death in kidney biopsies during cold storage. (a) Representative images of left and right kidneys stained with TUNEL assay after 6 or 72 h on cold storage. Overlays show area quantified after training DigiPath to recognize TUNEL‐positive (brown) or TUNEL‐negative (blue) cells. Scale bars represent 50 μm. (b) Quantification TUNEL staining in left and right kidneys at the beginning and end of cold storage using DigiPath. In (b), the ratio of TUNEL‐positive (brown) cell area to TUNEL‐negative (blue) cell area is plotted. (c) Representative images from immunofluorescence TUNEL staining are presented (red—TUNEL; blue—DAPI). Scale bars represent 50 μm. (d) Quantification of TUNEL staining in left and right kidneys at the beginning and end of cold storage using immunofluorescence. In (d), the number of TUNEL positive cells are divided by the number of DAPI cells. Each dot represents one field of view within the biopsy. Lines represent the median. ****p < 0.0001

To evaluate DigiPath in a novel application, we assessed the degree of cell death in non‐transplanted human kidneys during the course of cold storage (Table 2). Based on findings in a recently completed study, we hypothesized that cell death may be occurring during the course of cold storage in some marginal human organs.^9^ We applied TUNEL staining to biopsies collected from a pair of kidneys after 6, 12, 18, 24, 30, 36, 48, 60 and 72 h of cold storage (Figure 5). DigiPath was able to detect both TUNEL‐positive (brown) and TUNEL‐negative (blue) cells (nuclei) in each image (Figure 5a). This enabled us to quantify the amount of TUNEL staining normalized to the total cell density in each image (Figure 5b).

We observed a significant increase in TUNEL staining area normalized to the TUNEL negative cell area from 6 to 72 h of cold storage in both kidneys (Figure 5b). The left kidney had a greater proportion of TUNEL positive cells than the right kidney at 72 h (left median: 0.16 brown: blue area ratio; right median: 0.11 brown: blue area ratio; *p* < 0.0001) We found that quantification of dead cells in ImageJ using immunofluorescent TUNEL staining yielded similar trends. At 6 h of cold storage we observed <1% TUNEL positive cells. At 72 h of cold storage, we quantified a median of 62.4% and 13.7% TUNEL positive cells in the left and right kidneys respectively (Figure 5c,d). We then used DigiPath to quantify TUNEL staining in biopsies taken at intermediate time points and found that both organs had a small early spike in cell death at 12–18 h, followed by a larger increase in cell death beginning at 30–36 h and continuing up to 72 h (Supplemental Figure 4).

## 3 DISCUSSION

Quantitative analysis of histological samples poses a significant challenge due to the nature of color images as an overlay of red, green, and blue channels. While tissues stained with immunofluorescence can be evaluated by measuring each target in its individual color channel, nearly all features in a color image are made up of a mixture of three colors. As a result, histological features that are easily identifiable by eye are difficult to encode as a set of computational rules that an automated program can use to accurately detect features. Despite the difficulty of generating quantitative results from histology slides, they remain an important and highly used element of preclinical research.

To address this need, we have developed the DigiPath program which is a color‐based classification algorithm that facilitates rapid and reliable quantification of histology features. DigiPath can be used to quantify stained features in any RGB color image. It is particularly advantageous in preclinical research settings with large sample numbers, as it enables high‐throughput, quantitative assessment of unlimited numbers of high‐resolution images. To quantify features of interest in the biopsies presented here, we captured 20× fields of view covering the entire biopsy. This resulted in up to 400 images per biopsy, depending on the biopsy size. In this study, we analyzed 25 whole biopsies for a total of over 1800 images. Assessing this quantity of data by hand is simply not feasible regardless of access to a trained pathologist. In addition, DigiPath also allows researchers to access more nuanced information about the spatial heterogeneity of pathologic features. This added information can enable a more comprehensive characterization of pathologies (e.g., hepatic steatosis) both within and between biopsies.

DigiPath is a classification algorithm that makes decisions based directly on the categorization of pixel colors assigned during the training as “positive” or “negative.” Since a typical image contains millions of pixels, selecting positive and negative regions on a few images generates classification data from hundreds of thousands of pixels. This approach allows a large training set of pixel color data points to be generated from a relatively small number of training images and further enables rapid implementation of DigiPath into a research workflow. DigiPath is therefore distinct from more broadly encompassing machine learning approach which require extensive training data sets of manually annotated images in order to account for all possible staining variability. However, for this approach to be effective, all images to be analyzed must share the same basic color palate. Thus, we recommend that users run a new training set for each batch of samples to be analyzed; this ensures consistency in the color of pathologic features between the training images and images intended for quantitative analysis.

There are some limitations of DigiPath in its current iteration. As noted, applications of DigiPath are currently limited to features that can be defined solely by color. The DigiPath user‐interface provides options for filtering features by size; however, histological analyses that rely solely on morphology and do not employ distinguishing color stains cannot be performed using DigiPath. Hands‐on analysis time using DigiPath is greatly reduced relative to hand tracing; however, hands‐on time may increase if samples collected in a study were stained on different days or in different facilities, likely requiring separate training runs for each biopsy. While many potential sources of color variation can be controlled within a study (e.g., image collection settings, tissue thickness, staining procedures), there may be some experimental variables that introduce differences in staining between samples that cannot be avoided. For example, some features of interest may have distinct histological appearances in different species, meaning DigiPath results may not be directly comparable between species. Nevertheless, DigiPath can accommodate many other variable image properties including different tissue thicknesses and different magnification levels, as long as the training image set is consistent with the images to be analyzed. DigiPath can also analyze images of any size, depending on the memory limits of the user's computer. This is comparable to other commonly used image analysis software such as ImageJ.

Recent publications have described automated color image analysis methods developed for specific applications, including blood vessel segmentation and determination of differentiation potential of mesenchymal stem cells.^14^, ^16^ Other automated color image analysis programs have been developed to detect specific stains, such as diaminobenzidine.^15^ Like DigiPath, these examples detect features based on color. However, DigiPath's versatility allows a researcher to use a single tool for a broad range of color‐based quantification applications. There are a number of programs with image analysis tools available for download. ImageJ (imagej.nih.gov/ij) is widely used in biomedical research to view, edit, and analyze both fluorescent and color images.^11^, ^13^, ^14^, ^15^, ^16^, ^19^, ^20^, ^21^, ^22^ In this study we used ImageJ's Color Threshold feature and found that this method consistently resulted in lower correlation with hand‐traced standards compared to DigiPath. QuPath is a recently developed open source image analysis software and a powerful tool for whole slide image analysis.^23^ Other free downloadable image analysis programs include CellProfiler (cellprofiler.org), BioImageXD (bioimagexd.net), and Advanced Cell Classifier (cellclassifier.org).^24^, ^25^, ^26^ However, the descriptions of these programs focus on applications for confocal or fluorescent images, while color image analysis is not emphasized, if described at all. To our knowledge, there are no comparable open‐access programs available for color image analysis. Thus, we believe DigiPath's adaptability, ease of use, and transparent classification algorithm make it a useful tool for preclinical researchers seeking rapid quantitative analysis of histologic samples.

## 4 MATERIALS AND METHODS

### 4.1 Organ retrieval and storage

All non‐transplanted organs were provided under an existing research protocol with New England Donor Services (NEDS) after obtaining research consent from the donor families. In the United States, since the donor is deceased, this research is not considered human subject research as defined in the HHS Policy for Protection of Human Research Subjects 45 CFR 46.102. HIPAA is still invoked with these specimens and all organs used have been de‐identified. After in situ flushing of the abdominal organs with either cold University of Wisconsin or custodial histidine‐tryptophan‐ketoglutarate (HTK) preservation solution, organs were procured, packed in ice, and placed in static cold storage prior to experimentation per standard clinical practice.

### 4.2 Kidney normothermic machine perfusion

Kidneys were prepared and perfused for 1 h on the ex vivo normothermic machine perfusion circuit as previously described.^27^ Biopsies were collected at the end of the perfusion period.

### 4.3 Biopsy and staining procedures

Wedge biopsies were collected and fixed in 10% formalin for a minimum of 24 h. Formalin fixed biopsies were paraffin embedded, sectioned to 4 μm, and stained with either H&E, Sirius red, martius scarlet blue (MSB), or terminal deoxynucleotidyl transferase dUTP nick end labeling (TUNEL) stain in the Yale Histology laboratory. Immunofluorescent TUNEL staining was performed on 6 μm cryosections using the In Situ Cell Death Detection Kit, TMR red (Sigma).

### 4.4 Mouse model of steatohepatitis

C57BL/6J mice were from the National Cancer Institute as previously described.^18^ All experiments were performed in specific pathogen‐free facilities and were performed in accordance with the regulations adopted by the National Institutes of Health (NIH) and approved by the Animal Care and Use Committee of the Yale University. Eight‐week old male C57BL/6J mice were placed on a high‐fat diet (HFD; 45% fat, D12451 Research Diets) or chow as a model for steatohepatitis and chronic liver injury. After 10 weeks of a HFD animals were given a vehicle or digoxin at multiple dosages (low 0.125 mg/kg, medium 0.5 mg/kg, high 2.5 mg/kg) twice a week by gavage feeding and continued with HFD feeding for a total of 15 weeks. At the end of the protocols, whole livers were collected for histological analysis and stained with H&E. Images were collected at 10× magnification in the Ouyang Laboratory.

### 4.5 Brightfield imaging

Three sections per biopsy were tiled at 20× magnification using an EVOS FL Auto 2 microscope (ThermoFischer Scientific). All new images were captured as 24‐bit RGB color images with 3.2 million pixels (12 MB) at a resolution of 58,522 pixels per inch. Following image collection, images were manually parsed into “edge” versus “continuous” images to distinguish images that were wholly contained within the section (continuous) versus images that were partially tissue and partially blank space (edge). Edge images were excluded to avoid artifacts in analysis. Continuous images were then loaded into the program for quantification.

### 4.6 Hand tracing analysis

Users generating the hand tracing results were given explicit instructions on how to use the Freehand selections tool in ImageJ. While hand tracing each image, users recorded their screens for accurate measurements of the time it took to do each image analysis. After hand tracing each image, users measured the areas and created binary masks of their outlines.

### 4.7 Color threshold analysis

Images were analyzed in ImageJ. The “Threshold Color” window was opened, and a set of red, green, and blue thresholds was chosen in red/green/blue (RGB) space using the default thresholding method. The selection was converted to a binary mask, which was saved for evaluating accuracy relative to hand tracing.

### 4.8 DigiPath program

The DigiPath custom program was developed in MATLAB (Version R2019b; The Mathworks, Inc. 2019) with the Image Processing Toolbox (The Mathworks, Inc. 2020) installed. The complete code, entitled “DigiPath.mlapp,” is available as a packaged app on Mathworks.com, and in the Supplemental Materials.

### 4.9 Image analysis using custom MATLAB program

After eliminating edge images from each experimental set of 24‐bit color images, two to four training images that were representative of any variations in staining were selected by the user. The images analyzed in this study had 800,000 pixels (3 MB per image; Figures 1 and 2), 1.4 million pixels (5.3 MB per image; Figure 4), or 3.2 million pixels (12 MB per image; Figures 3 and 5). The program was initiated, and positive and negative regions were selected as prompted by drawing polygons of any shape and size on the displayed training images. The complete image set was run through the algorithm using the color map and positive color list generated in the training steps. Binary mask overlays of regions the program had identified as positively stained were reviewed visually. Images for which the program‐determined regions did not line up with visually identifiable features were run through the program a second time using an updated color map and positive color list. Results are represented by plotting the percent of the image area with positive staining. Each point represents one image field within a biopsy. The significance of differences between biopsies was calculated using one‐way ANOVA.

### 4.10 Evaluation of DigiPath performance

Area of microvascular obstructions was quantified in six 20× images of kidneys stained with H&E using three quantitative methods: hand tracing, color thresholding, and DigiPath analysis. A set of three independent users analyzed the same six images using each method. A consensus hand‐traced standard was generated for each image by including areas that were selected by all three users. This was used as a standard for comparison to areas found by color thresholding or DigiPath. A confusion matrix was generated where the hand‐traced classification of the images served as the “true” positive and negative values, and the classification by color thresholding or DigiPath were positioned as the “predicted” positive and negative values. Metrics including sensitivity, specificity, and accuracy were calculated from each matrix. Three correlation coefficients, F‐score, Matthew's correlation coefficient (MCC), and Youden's J statistic, were calculated to assess the performance of the color thresholding and DigiPath methods in the hands of each independent user. The significance of differences between methods was calculated using two‐way ANOVA.

### 4.11 Correlation metrics

Three correlation metrics were derived from a confusion matrix of all possible classification outcomes (FP—false positives, FN—false negatives, TP—true positives, and TN—true negatives). Youden's J statistic represents “informedness”—the probability that the program will make an informed decision.^28^ This takes into account both sensitivity and specificity.

$$J=\frac{\mathrm{TP}}{\mathrm{TP}+\mathrm{FN}}+\frac{\mathrm{TN}}{\mathrm{TN}+\mathrm{FP}}-1=\text{sensitivity}+\text{specificity}-1.$$

The F‐score is a measure of accuracy and is derived from sensitivity and precision values.^29^

$$F=\frac{2\mathrm{TP}}{2\mathrm{TP}+\mathrm{FP}+\mathrm{FN}}=2\times \frac{\text{precision}\times \text{sensitivity}}{\text{precision}+\text{sensitivity}}.$$

MCC is a measure of both markedness and informedness and accounts for the proportion of occurrences of each possible classification outcome.^17^, ^30^, ^31^

$$\mathrm{MCC}=\frac{\mathrm{TP}\times \mathrm{TN}-\mathrm{FP}\times \mathrm{FN}}{\sqrt{\left(\mathrm{TP}+\mathrm{FP}\right)\left(\mathrm{TP}+\mathrm{FN}\right)\left(\mathrm{TN}+\mathrm{FP}\right)\left(\mathrm{TN}+\mathrm{FN}\right)}}.$$

### 4.12 ImageJ quantification

Immunofluorescent TUNEL images were processed using the Watershed and Analyze Particles functions in ImageJ. The total number of DAPI positive (blue) and TUNEL positive (red) cells were quantified.

## 5 CONCLUSIONS

DigiPath is a highly adaptable tool that enables high‐throughput, quantitative analysis of any color‐defined histologic feature. DigiPath is available for download as a free app in the MATLAB File Exchange (www.mathworks.com/matlabcentral/fileexchange). The app is accessible to researchers regardless of their level of experience with coding and can be operated by users who are not formally trained in pathology. The ability to automatically detect features in histology images based on three‐channel RGB color data enables a more quantitative approach to histological analysis, an experimental technique that is already essential in preclinical research.

## CONFLICT OF INTEREST

The authors have no conflict of interest to declare.

## AUTHOR CONTRIBUTIONS

**Melanie Reschke:** Conceptualization; data curation; formal analysis; investigation; methodology; project administration; validation; visualization; writing ‐ original draft; writing‐review & editing. **Jenna DiRito:** Conceptualization; data curation; formal analysis; investigation; methodology; project administration; validation; visualization; writing ‐ original draft; writing‐review & editing. **David Stern:** Formal analysis; validation. **Wesley Day:** Formal analysis; validation. **Natalie Plebanek:** Formal analysis; validation. **Matthew Harris:** Data curation. **Sarah Hosgood:** Supervision; writing‐review & editing. **Michael Nicholson:** Supervision; writing‐review & editing. **Danielle Haakinson:** Data curation; writing‐review & editing. **Xuchen Zhang:** Data curation; formal analysis; writing‐review & editing. **Wajahat Mehal:** Data curation; resources; writing‐review & editing. **Xinshou Ouyang:** Data curation; resources; writing‐review & editing. **Jordan Pober:** Conceptualization; funding acquisition; methodology; supervision; writing‐review & editing. **W Saltzman:** Conceptualization; funding acquisition; methodology; supervision; writing‐review & editing. **Gregory Tietjen:** Conceptualization; formal analysis; methodology; project administration; resources; supervision; validation; visualization; writing ‐ original draft; writing‐review & editing.

### PEER REVIEW

The peer review history for this article is available at https://publons.com/publon/10.1002/btm2.10242.

## Supporting information


**Appendix** **S1**: Supporting Information

**Appendix** **S2**: Supporting Information

**Supplemental Figure 1**: Hand tracing overlines areas of interest compared to DigiPath. Overlays of hand‐traced (blue) or DigiPath (green) mask on original H&E obstruction (red). Orange box defines area of interest.
**Supplemental Figure 2**: Analysis time with DigiPath and hand tracing. (A) Estimated extrapolation of analysis time for hand tracing (black line) or DigiPath (red line) in larger imaging sets of up to 500 images.
**Supplemental Figure 3**: DigiPath app user interface. The app interface is organized into three sections. The “Settings” section is used to select image folders and file types, assign the number of training images, and choose whether to exclude background areas. The “Run Program” section is used to initiate the training portion of the workflow or load previously generated training data, and to run the automated quantification once the training portion is complete. The “Advanced Settings” section (toggled to visible/invisible) is a panel with additional options to customize aspects of the analysis such as size thresholds, mask color, and morphological operations.
**Supplemental Figure 4**: Kinetics of cell death during cold storage. Quantification of TUNEL staining in Left (top) and Right (bottom) kidneys during cold storage. The ratio of TUNEL‐positive (brown) cell area to TUNEL‐negative (blue) cell area is plotted. Each dot represents one field of view within the biopsy. Lines represent the median. **p < 0.01; ***p < 0.001; ****p < 0.0001.

## Data Availability

The data that support the findings of this study are available from the corresponding author upon reasonable request.
