# Supplementary material for: A digital pathology tool for quantification of color features in histologic specimens
Source: Bioeng Transl Med. 2021 Aug 24;7(1):e10242. doi: 10.1002/btm2.10242 (PMC8780932; doi:10.1002/btm2.10242)
Supplement: Supplementary file 1 — Appendix S1: Supporting Information [file BTM2-7-e10242-s001.docx]

**Supplementary Methods:**

The DigiPath program is operated through a user-interface with interactive fields to select the image folder location, define a file-name filter if needed, enter the desired number of training images, and select whether image fields contain background to be cropped out. Click-buttons initiate the training portion and the automated analysis portion, and an interactive image window displays the training images sequentially. An ‘Advanced Settings’ panel enables additional customization of the code including defining the range of object sizes to be detected, enabling additional morphological operations, and customizing the output image style.

Upon initiation of the training portion, the program prompts the user to select areas that are either ‘positive’ or ‘negative’ based on color staining by clicking to outline regions on the training images. The algorithm then compares pixel colors found in both positive and negative regions to generate a list of pixel colors that are only found in regions identified as positive (Positive Color List). This list, along with a list of all colors present in the training images (Color Map), is saved for immediate or future use in the automated portion of the application.

When the automated portion is initiated, the algorithm iterates through all images in the selected folder. As each image is loaded for analysis, the Color Map is applied to define all colors present in the image. Each pixel in the image is then compared to the Positive Color List. A new binary mask linked to each image is generated, where colors consistent with the Positive Color List are marked ‘True’, and colors that are not contained in the list are ‘False’. Morphological operations of dilation, erosion, and hole-filling (optional) are performed to smooth out positive objects. A size filter is applied based on the default (50 pixels) or a custom user input minimum object size. This erases small points that may be considered noise. The program then sums and stores the value of the number of positive pixels in the binary matrix. The binary matrix is used to generate and save a figure of the original color image with a mask overlay showing the positively identified regions. After all of the images in the folder have been analyzed, the program outputs a spreadsheet containing the image name, number of positive pixels, total pixels in the image (or tissue, if background area has been subtracted), and a percentage of positive staining over total tissue area.
