# Supplementary material for: A digital pathology tool for quantification of color features in histologic specimens
Source: Bioeng Transl Med. 2021 Aug 24;7(1):e10242. doi: 10.1002/btm2.10242 (PMC8780932; doi:10.1002/btm2.10242)
Supplement: Supplementary file 2 — Appendix S2: Supporting Information [file BTM2-7-e10242-s002.rtf]

classdef DigiPath < matlab.apps.AppBase    % Properties that correspond to app components    properties (Access = public)        UIFigure                        matlab.ui.Figure        ImageAxes                       matlab.ui.control.UIAxes        ShowAdvancedSettingsButton      matlab.ui.control.StateButton        Panel                           matlab.ui.container.Panel        Minimumobjectsizepixelsdefault50EditFieldLabel  matlab.ui.control.Label        Minimumobjectsizepixelsdefault50EditField  matlab.ui.control.NumericEditField        Minbackgroundareasizepixelsdefault100000EditFieldLabel  matlab.ui.control.Label        Minbackgroundareasizepixelsdefault100000EditField  matlab.ui.control.NumericEditField        FillholesdefaultOffLabel        matlab.ui.control.Label        FillholesdefaultOffSwitch       matlab.ui.control.Switch        MaskDisplayColorDropDownLabel   matlab.ui.control.Label        MaskDisplayColorDropDown        matlab.ui.control.DropDown        AdvancedSettingsLabel           matlab.ui.control.Label        Morphologicalstructuringelementradiusdefault3Label  matlab.ui.control.Label        Morphologicalstructuringelementradius  matlab.ui.control.Spinner        Panel_2                         matlab.ui.container.Panel        RunTrainingImagesButton         matlab.ui.control.Button        RunQuantificationButton         matlab.ui.control.Button        LoadTrainingDataButton          matlab.ui.control.Button        ORLabel                         matlab.ui.control.Label        Label_2                         matlab.ui.control.Label        Label_3                         matlab.ui.control.Label        RunProgramLabel                 matlab.ui.control.Label        EditField_3                     matlab.ui.control.EditField        Panel_3                         matlab.ui.container.Panel        SelectImageFolderButton         matlab.ui.control.Button        EditField                       matlab.ui.control.EditField        EnterFileNameFilteroptionalEditFieldLabel  matlab.ui.control.Label        EnterFileNameFilteroptionalEditField  matlab.ui.control.EditField        canfillinforanynumberofcharactersLabel  matlab.ui.control.Label        SelectResultDestinationFolderButton  matlab.ui.control.Button        EditField_2                     matlab.ui.control.EditField        EnterNumberofTrainingImagesLabel  matlab.ui.control.Label        EnterNumberofTrainingImagesEditField  matlab.ui.control.NumericEditField        Label                           matlab.ui.control.Label        SubtractBackgroundAreaCheckBox  matlab.ui.control.CheckBox        SettingsLabel                   matlab.ui.control.Label    end        methods (Access = private)                function updateImage(app,displayImage)                                    app.ImageAxes.XLimMode = 'auto';            app.ImageAxes.YLimMode = 'auto';            imshow(displayImage, 'Parent', app.ImageAxes);            axtoolbar(app.ImageAxes,{'zoomin','zoomout','restoreview'},'Visible','on');            app.ImageAxes.Interactions = [zoomInteraction];            enableDefaultInteractivity(app.ImageAxes);        end                function h = subplot_threePanel(app,n,m,i)            [c,r] = ind2sub([m n],i);            ax = subplot('Position',[(c-1)/m, 1-(r)/n, 1/m, 1/n]);            if (nargout>0)                h=ax;            end        end            end        % Callbacks that handle component events    methods (Access = private)        % Button pushed function: RunTrainingImagesButton        function RunTrainingImagesButtonPushed(app, event)                        % Get image folder path            imagesPath = app.EditField.Value;            FileFilter = app.EnterFileNameFilteroptionalEditField.Value;            fullFile = fullfile(imagesPath, FileFilter);            images = dir(fullFile);                        savePath = app.EditField_2.Value;            app.EditField_3.Value = savePath;            addpath(app.EditField_3.Value);                        % Choose training images based on input number and number of            % files in folder            numTrainingImages = app.EnterNumberofTrainingImagesEditField.Value;            numTotImages = length(images);            trainingImageInterval = floor(numTotImages/numTrainingImages);                        % Based on how many training images are selected, define the            % dimensions of an array of training images (assume square layout of images)            trainingImageIdx = zeros(1,numTrainingImages);            for pp = 1:numTrainingImages                    trainingImageIdx(pp) = (pp*trainingImageInterval) - trainingImageInterval +1;            end            stitchedTrainingDims = ceil(sqrt(length(trainingImageIdx)));            trainingImages = cell(1,numTrainingImages);            stitchedTrainingCell = cell(stitchedTrainingDims);                        % Store Training Images in Cell Array            for ll = 1:length(trainingImageIdx)                                n = trainingImageIdx(ll);                clear img                    img_name   = images(n).name;                    img.fourdim   = imread(img_name);                img.ui8 = img.fourdim(:,:,1:3);                     trainingImages{ll} = img.ui8;              end            img.height = size(img.ui8,1);            img.width  = size(img.ui8,2);            whiteimgfill = uint8((zeros(img.height,img.width,3)+1)*2^8);            % Arrange the training images, still in cells, into a square            % arrangement. any leftover cells in the square will be            % filled with white images            nn=0;            for Row = 1:stitchedTrainingDims                for Col = 1:stitchedTrainingDims                    nn=nn+1;                    if nn <= length(trainingImages)                        stitchedTrainingCell{Row,Col} = trainingImages{nn};                    else                        stitchedTrainingCell{Row,Col} = whiteimgfill;                    end                end            end                        % Convert the square layout cell array into a matrix, so it is            % one big ui8 image, and image is displayed in app            stitchedTraining = cell2mat(stitchedTrainingCell);            updateImage(app,stitchedTraining);                        % Wait for confirmation of training images            h = msgbox('Confirm Training Images');            uiwait(h);                        % Generate Color Map from Stitched Training Images                    [~,map] = rgb2ind(stitchedTraining,65536); %second input in this function is the number of colors. will run faster with fewer colors but you will get more false negative pixels            uniqueMM=unique(map,'rows');                                    % Begin selection of regions in training images            storedPosColors = zeros(length(uniqueMM),length(trainingImageIdx));            storedBgroundColors = zeros(length(uniqueMM),length(trainingImageIdx));            for ii = trainingImageIdx                img_name = images(ii).name;                img.fourdim   = imread(img_name);                img.ui8 = img.fourdim(:,:,1:3);                    updateImage(app,img.ui8);                                figure(app.UIFigure);                    % select first positive region                h = msgbox('Select positive region 1');                uiwait(h);                findposreg1 = drawpolygon(app.ImageAxes,'Color','blue');                img.findposreg1 = createMask(findposreg1);                                % select second positive region                h = msgbox('Select positive region 2');                uiwait(h);                findposreg2 = drawpolygon(app.ImageAxes,'Color','blue');                img.findposreg2 = createMask(findposreg2);                                % select third positive region                h = msgbox('Select positive region 3');                uiwait(h);                findposreg3 = drawpolygon(app.ImageAxes,'Color','blue');                img.findposreg3 = createMask(findposreg3);                    % make array where 1 is in selected positive regions and 0 is not                img.index_pos = img.findposreg1 + img.findposreg2 + img.findposreg3;                        % make index of the linear index of selected positive pixels                idx_pos = sort(unique(cat(1,find(img.findposreg1),find(img.findposreg2),find(img.findposreg3))));                                % select first negative region                h = msgbox('Select negative region 1');                uiwait(h);                findnegreg1 = drawpolygon(app.ImageAxes,'Color','red');                img.findnegreg1 = createMask(findnegreg1);                    % select second negative region                h = msgbox('Select negative region 2');                uiwait(h);                findnegreg2 = drawpolygon(app.ImageAxes,'Color','red');                img.findnegreg2 = createMask(findnegreg2);                                % select third negative region                h = msgbox('Select negative region 3');                uiwait(h);                findnegreg3 = drawpolygon(app.ImageAxes,'Color','red');                img.findnegreg3 = createMask(findnegreg3);                                % make array where 1 is in selected negative regions and 0 is not                img.index_neg = img.findnegreg1 + img.findnegreg2 + img.findnegreg3;                        % make index of the linear index of selected negative pixels                idx_neg = sort(unique(cat(1,find(img.findnegreg1),find(img.findnegreg2),find(img.findnegreg3))));                                                if app.SubtractBackgroundAreaCheckBox.Value == 1                    h = msgbox('Select Slide Background Region');                        uiwait(h);                        findbgroundreg = drawpolygon(app.ImageAxes,'Color','green');                        img.findbgroundreg = createMask(findbgroundreg);                                                img.index_bground = img.findbgroundreg;                        idx_bground = sort(unique(cat(1,find(img.findbgroundreg))));                end                                % convert to an indexed image with a colormap, display image                X = rgb2ind(img.ui8,uniqueMM);                                % find all colors from colormap in both positive and negative selected                % regions                poscolors = double(sort(unique(X(idx_pos))));                negcolors = double(sort(unique(X(idx_neg))));                                if app.SubtractBackgroundAreaCheckBox.Value == 1                    bgroundcolors = double(sort(unique(X(idx_bground))));                    storedBgroundColors(1:length(bgroundcolors),ii) = bgroundcolors;                end                    % make a list of all colors that                 nooverlap = poscolors(logical(-1*(ismember(poscolors,negcolors) - 1)));                                            storedPosColors(1:length(nooverlap),ii) = nooverlap;            end                       PosColorList = unique(nonzeros(reshape(storedPosColors,[],1)));                        PosColorListName_mat = sprintf('PosColorList.mat');            RefColorMapName_mat = sprintf('RefColorMap.mat');                        save(fullfile(savePath,PosColorListName_mat),'PosColorList');             save(fullfile(savePath,RefColorMapName_mat),'uniqueMM');                        if app.SubtractBackgroundAreaCheckBox.Value == 1                BackgroundColorList = unique(nonzeros(reshape(storedBgroundColors,[],1)));                BackgroundColorListName_mat = sprintf('BackgroundColors.mat');                save(fullfile(savePath,BackgroundColorListName_mat),'BackgroundColorList');            end                        updateImage(app,stitchedTraining);            h = msgbox('Training Complete');            uiwait(h);            figure(app.UIFigure);        end        % Value changed function:         % EnterFileNameFilteroptionalEditField        function EnterFileNameFilteroptionalEditFieldValueChanged(app, event)                    end        % Button pushed function: SelectImageFolderButton        function SelectImageFolderButtonPushed(app, event)            f = uifigure('Name', 'Opening File Browser');            drawnow;            f.Visible = 'off';            app.EditField.Value = uigetdir;            delete(f);            clear f;            figure(app.UIFigure);            addpath(app.EditField.Value);            app.EditField.FontColor = 'black';        end        % Button pushed function: RunQuantificationButton        function RunQuantificationButtonPushed(app, event)            % Get image folder path            imagesPath = app.EditField.Value;            FileFilter = app.EnterFileNameFilteroptionalEditField.Value;            fullFile = fullfile(imagesPath, FileFilter);            images = dir(fullFile);                        savePath = app.EditField_2.Value;                        PosColorListName_mat = sprintf('PosColorList.mat');            RefColorMapName_mat = sprintf('RefColorMap.mat');                        load(fullfile(app.EditField_3.Value,PosColorListName_mat));            load(fullfile(app.EditField_3.Value,RefColorMapName_mat));            if app.SubtractBackgroundAreaCheckBox.Value == 1                 BackgroundColorListName_mat = sprintf('BackgroundColors.mat');                 load(fullfile(app.EditField_3.Value,BackgroundColorListName_mat));            end                                                Results(1,1)= {'Image Name'};            Results(1,2)= {'Positive Area (pixels)'};            Results(1,3)= {'Region Area (pixels)'};            Results(1,4)= {'Background Area (pixels)'};            Results(1,5)= {'Percent Positive Area (%)'};            for ii = 1:length(images)                img_name = images(ii).name;                [~,name,~] = fileparts(images(ii).name);                img.fourdim   = imread(img_name);                img.ui8 = img.fourdim(:,:,1:3);                img.height = size(img.ui8,1);                img.width = size(img.ui8,2);                                indimage = dither(img.ui8,uniqueMM,8,7);                                if app.SubtractBackgroundAreaCheckBox.Value == 1                                        indimage_bgroundMask = ismember(indimage,BackgroundColorList);                                        SE = strel('disk',3,4);                    dilatedBground = imdilate(indimage_bgroundMask,SE);                    erodedBground = imerode(dilatedBground,SE);                    filledBWmaskBground = erodedBground;                                        conncompBground = bwconncomp(filledBWmaskBground);                    numPixelsBground = cellfun(@numel,conncompBground.PixelIdxList);                    img.Bground = zeros(img.height, img.width);                      for kk = 1:conncompBground.NumObjects                          if numPixelsBground(kk) > app.Minbackgroundareasizepixelsdefault100000EditField.Value                             img.Bground(conncompBground.PixelIdxList{kk}) = 1;                          end                      end                                        img.findreg = -1*(img.Bground-1);                                        bground_pixelArea = nnz(img.Bground);                                         filledbgroundmask = uint8(img.Bground*2^8);                    filledbluebgroundmask = cat(3,zeros(img.height,img.width),zeros(img.height,img.width),filledbgroundmask);                                        imagebgroundMaskSubtracted = img.ui8-filledbgroundmask;                                        updateImage(app,filledbluebgroundmask+imagebgroundMaskSubtracted);                                        fig = figure('Visible','off');                    fig.Units = 'normalized';                    fig.OuterPosition = [0 0.2 0.8 0.8];                                        subplot_threePanel(app,1,3,1), imshow(filledbluebgroundmask+imagebgroundMaskSubtracted);                    subplot_threePanel(app,1,3,2), imshow(filledbluebgroundmask);                    subplot_threePanel(app,1,3,3), imshow(img.ui8);                                        bgroundpicturename = sprintf([name '_slide_background']);                    bgroundmaskpicturename = sprintf([name '_background_mask.tiff']);                                        saveas(gcf,fullfile(savePath, bgroundpicturename),'tiffn');                     delete(fig);                     clear fig;                                        imwrite(filledbluebgroundmask,fullfile(savePath, bgroundmaskpicturename));                                else                    img.findreg = zeros(img.height,img.width)+1;                    bground_pixelArea = 0;                end                                reg_pixelArea = nnz(img.findreg);                                indimage_posMask = ismember(indimage,PosColorList); %makes binary: 1 if positive, 0 if negative, for whole image(not ROI specific)                indimage_posMaskReg = indimage_posMask.*img.findreg;                                  % dilate, erode ,fill holes in mask                SE = strel('disk',app.Morphologicalstructuringelementradius.Value,4);                dilated = imdilate(indimage_posMaskReg,SE);                eroded = imerode(dilated,SE);                                      if strcmp(app.FillholesdefaultOffSwitch.Value,'On') == 1                    filledBWmask = imfill(eroded,'holes');                else                    filledBWmask = eroded;                end                conncomp = bwconncomp(filledBWmask);                numPixels = cellfun(@numel,conncomp.PixelIdxList);                img.stain = zeros(img.height, img.width);                  for kk = 1:conncomp.NumObjects                      if numPixels(kk) > app.Minimumobjectsizepixelsdefault50EditField.Value                         img.stain(conncomp.PixelIdxList{kk}) = 1;                      end                  end                                      % calculate the positive pixel number                pos_pixelArea = nnz(img.stain);                                filledmask = uint8(img.stain*2^8);                                                if strcmp(app.MaskDisplayColorDropDown.Value,'Green') == 1                    filledColorMask = cat(3,zeros(img.height,img.width),filledmask,zeros(img.height,img.width));                elseif strcmp(app.MaskDisplayColorDropDown.Value,'Blue') == 1                      filledColorMask = cat(3,zeros(img.height,img.width),zeros(img.height,img.width),filledmask);                elseif strcmp(app.MaskDisplayColorDropDown.Value,'Red') == 1                        filledColorMask = cat(3,filledmask,zeros(img.height,img.width),zeros(img.height,img.width));                elseif strcmp(app.MaskDisplayColorDropDown.Value,'Cyan') == 1                    filledColorMask = cat(3,zeros(img.height,img.width),filledmask,filledmask);                elseif strcmp(app.MaskDisplayColorDropDown.Value,'Magenta') == 1                    filledColorMask = cat(3,filledmask,zeros(img.height,img.width),filledmask);                elseif strcmp(app.MaskDisplayColorDropDown.Value,'Yellow') == 1                    filledColorMask = cat(3,filledmask,filledmask,zeros(img.height,img.width));                end                                    imageMaskSubtracted = img.ui8-filledmask;                                updateImage(app,filledColorMask+imageMaskSubtracted);                                fig = figure('Visible','off');                fig.Units = 'normalized';                fig.OuterPosition = [0 0.2 0.8 0.8];                 subplot_threePanel(app,1,3,1), imshow(filledColorMask+imageMaskSubtracted);                subplot_threePanel(app,1,3,2), imshow(filledColorMask);                subplot_threePanel(app,1,3,3), imshow(img.ui8);                                % save images                picturename = sprintf([name '_detected_stain']);                maskpicturename = sprintf([name '_mask.tiff']);                              saveas(gcf,fullfile(savePath, picturename),'tiffn');                 delete(fig);                 clear fig;                            imwrite(filledColorMask,fullfile(savePath, maskpicturename));                                % store image name, positive pixels, total pixels in Results table                Results(ii+1,1) = {img_name};                Results(ii+1,2) = {pos_pixelArea};                Results(ii+1,3) = {reg_pixelArea};                Results(ii+1,4) = {bground_pixelArea};                Results(ii+1,5) = {100*(pos_pixelArea/reg_pixelArea)};                             end            ResultsName_mat = sprintf('Results.mat');            ResultsName_csv = sprintf('Results.csv');            ResultsName_xls = sprintf('Results.xls');            save(fullfile(savePath,ResultsName_mat),'Results');            writecell(Results, fullfile(savePath,ResultsName_csv));            writecell(Results, fullfile(savePath,ResultsName_xls));            figure(app.UIFigure);            h = msgbox('Quantification Complete');            uiwait(h);            figure(app.UIFigure);        end        % Value changed function: SubtractBackgroundAreaCheckBox        function SubtractBackgroundAreaCheckBoxValueChanged(app, event)            value = app.SubtractBackgroundAreaCheckBox.Value;                    end        % Value changed function: ShowAdvancedSettingsButton        function ShowAdvancedSettingsButtonValueChanged(app, event)            value = app.ShowAdvancedSettingsButton.Value;            if value == 1                app.Panel.Visible = 1;            end            if value == 0                app.Panel.Visible = 0;            end        end        % Value changed function:         % Minimumobjectsizepixelsdefault50EditField        function Minimumobjectsizepixelsdefault50EditFieldValueChanged(app, event)                    end        % Value changed function:         % Minbackgroundareasizepixelsdefault100000EditField        function Minbackgroundareasizepixelsdefault100000EditFieldValueChanged(app, event)                    end        % Callback function        function AdvancedSettingsSwitchValueChanged(app, event)                    end        % Value changed function: EditField        function EditFieldValueChanged(app, event)                    end        % Button pushed function:         % SelectResultDestinationFolderButton        function SelectResultDestinationFolderButtonPushed(app, event)            f = uifigure('Name', 'Opening File Browser');            drawnow;            f.Visible = 'off';            app.EditField_2.Value = uigetdir;               delete(f);            clear f;            figure(app.UIFigure);            addpath(app.EditField_2.Value);            app.EditField_2.FontColor = 'black';        end        % Value changed function: FillholesdefaultOffSwitch        function FillholesdefaultOffSwitchValueChanged(app, event)                    end        % Value changed function: MaskDisplayColorDropDown        function MaskDisplayColorDropDownValueChanged(app, event)                    end        % Callback function        function Morphologicalstructuringelementradiusdefault3SliderValueChanged(app, event)                    end        % Callback function        function MorphologicalstructuringelementradiusValueChanged(app, event)                    end        % Button pushed function: LoadTrainingDataButton        function LoadTrainingDataButtonPushed(app, event)            f = uifigure('Name', 'Opening File Browser');            drawnow;            f.Visible = 'off';            app.EditField_3.Value = uigetdir;            delete(f);            clear f;            figure(app.UIFigure);            addpath(app.EditField_3.Value);        end    end    % Component initialization    methods (Access = private)        % Create UIFigure and components        function createComponents(app)            % Create UIFigure and hide until all components are created            app.UIFigure = uifigure('Visible', 'off');            app.UIFigure.AutoResizeChildren = 'off';            app.UIFigure.Color = [1 1 1];            app.UIFigure.Position = [100 100 1080 810];            app.UIFigure.Name = 'UI Figure';            % Create ImageAxes            app.ImageAxes = uiaxes(app.UIFigure);            app.ImageAxes.Box = 'on';            app.ImageAxes.XTick = [];            app.ImageAxes.XTickLabel = {'[ ]'};            app.ImageAxes.YTick = [];            app.ImageAxes.LineWidth = 3;            app.ImageAxes.BackgroundColor = [1 1 1];            app.ImageAxes.Interruptible = 'off';            app.ImageAxes.Position = [213 7 855 597];            % Create ShowAdvancedSettingsButton            app.ShowAdvancedSettingsButton = uibutton(app.UIFigure, 'state');            app.ShowAdvancedSettingsButton.ValueChangedFcn = createCallbackFcn(app, @ShowAdvancedSettingsButtonValueChanged, true);            app.ShowAdvancedSettingsButton.Text = 'Show Advanced Settings';            app.ShowAdvancedSettingsButton.BackgroundColor = [0.8118 0.9608 0.9882];            app.ShowAdvancedSettingsButton.Position = [13 395 189 22];            % Create Panel            app.Panel = uipanel(app.UIFigure);            app.Panel.AutoResizeChildren = 'off';            app.Panel.Visible = 'off';            app.Panel.BackgroundColor = [0.8118 0.9608 0.9882];            app.Panel.Position = [13 13 189 353];            % Create Minimumobjectsizepixelsdefault50EditFieldLabel            app.Minimumobjectsizepixelsdefault50EditFieldLabel = uilabel(app.Panel);            app.Minimumobjectsizepixelsdefault50EditFieldLabel.Position = [7 224 121 39];            app.Minimumobjectsizepixelsdefault50EditFieldLabel.Text = {'Minimum object size'; '(pixels; default = 50):'};            % Create Minimumobjectsizepixelsdefault50EditField            app.Minimumobjectsizepixelsdefault50EditField = uieditfield(app.Panel, 'numeric');            app.Minimumobjectsizepixelsdefault50EditField.Limits = [0 Inf];            app.Minimumobjectsizepixelsdefault50EditField.RoundFractionalValues = 'on';            app.Minimumobjectsizepixelsdefault50EditField.ValueDisplayFormat = '%.0f';            app.Minimumobjectsizepixelsdefault50EditField.ValueChangedFcn = createCallbackFcn(app, @Minimumobjectsizepixelsdefault50EditFieldValueChanged, true);            app.Minimumobjectsizepixelsdefault50EditField.Position = [89 205 87 22];            app.Minimumobjectsizepixelsdefault50EditField.Value = 50;            % Create Minbackgroundareasizepixelsdefault100000EditFieldLabel            app.Minbackgroundareasizepixelsdefault100000EditFieldLabel = uilabel(app.Panel);            app.Minbackgroundareasizepixelsdefault100000EditFieldLabel.Position = [7 161 173 36];            app.Minbackgroundareasizepixelsdefault100000EditFieldLabel.Text = {'Min. background area size'; '(pixels; default = 100000):'};            % Create Minbackgroundareasizepixelsdefault100000EditField            app.Minbackgroundareasizepixelsdefault100000EditField = uieditfield(app.Panel, 'numeric');            app.Minbackgroundareasizepixelsdefault100000EditField.Limits = [0 Inf];            app.Minbackgroundareasizepixelsdefault100000EditField.RoundFractionalValues = 'on';            app.Minbackgroundareasizepixelsdefault100000EditField.ValueDisplayFormat = '%.0f';            app.Minbackgroundareasizepixelsdefault100000EditField.ValueChangedFcn = createCallbackFcn(app, @Minbackgroundareasizepixelsdefault100000EditFieldValueChanged, true);            app.Minbackgroundareasizepixelsdefault100000EditField.Position = [89 140 87 22];            app.Minbackgroundareasizepixelsdefault100000EditField.Value = 100000;            % Create FillholesdefaultOffLabel            app.FillholesdefaultOffLabel = uilabel(app.Panel);            app.FillholesdefaultOffLabel.Position = [7 88 132 57];            app.FillholesdefaultOffLabel.Text = 'Fill holes (default = Off):';            % Create FillholesdefaultOffSwitch            app.FillholesdefaultOffSwitch = uiswitch(app.Panel, 'slider');            app.FillholesdefaultOffSwitch.ValueChangedFcn = createCallbackFcn(app, @FillholesdefaultOffSwitchValueChanged, true);            app.FillholesdefaultOffSwitch.Position = [108 84 45 20];            % Create MaskDisplayColorDropDownLabel            app.MaskDisplayColorDropDownLabel = uilabel(app.Panel);            app.MaskDisplayColorDropDownLabel.Position = [7 291 113 23];            app.MaskDisplayColorDropDownLabel.Text = 'Mask Display Color:';            % Create MaskDisplayColorDropDown            app.MaskDisplayColorDropDown = uidropdown(app.Panel);            app.MaskDisplayColorDropDown.Items = {'Green', 'Blue', 'Red', 'Cyan', 'Magenta', 'Yellow'};            app.MaskDisplayColorDropDown.ValueChangedFcn = createCallbackFcn(app, @MaskDisplayColorDropDownValueChanged, true);            app.MaskDisplayColorDropDown.Position = [89 270 87 22];            app.MaskDisplayColorDropDown.Value = 'Green';            % Create AdvancedSettingsLabel            app.AdvancedSettingsLabel = uilabel(app.Panel);            app.AdvancedSettingsLabel.BackgroundColor = [0.6078 0.7882 0.8196];            app.AdvancedSettingsLabel.FontSize = 18;            app.AdvancedSettingsLabel.FontWeight = 'bold';            app.AdvancedSettingsLabel.Position = [0 326 188 26];            app.AdvancedSettingsLabel.Text = ' Advanced Settings';            % Create Morphologicalstructuringelementradiusdefault3Label            app.Morphologicalstructuringelementradiusdefault3Label = uilabel(app.Panel);            app.Morphologicalstructuringelementradiusdefault3Label.Position = [7 42 169 28];            app.Morphologicalstructuringelementradiusdefault3Label.Text = {'Morphological structuring'; 'element radius (default = 3):'};            % Create Morphologicalstructuringelementradius            app.Morphologicalstructuringelementradius = uispinner(app.Panel);            app.Morphologicalstructuringelementradius.Limits = [0 6];            app.Morphologicalstructuringelementradius.RoundFractionalValues = 'on';            app.Morphologicalstructuringelementradius.ValueDisplayFormat = '%.0f';            app.Morphologicalstructuringelementradius.Position = [89 15 87 22];            app.Morphologicalstructuringelementradius.Value = 3;            % Create Panel_2            app.Panel_2 = uipanel(app.UIFigure);            app.Panel_2.AutoResizeChildren = 'off';            app.Panel_2.ForegroundColor = [0.149 0.149 0.149];            app.Panel_2.BackgroundColor = [1 0.8549 0.5216];            app.Panel_2.FontWeight = 'bold';            app.Panel_2.FontSize = 16;            app.Panel_2.Position = [13 446 189 151];            % Create RunTrainingImagesButton            app.RunTrainingImagesButton = uibutton(app.Panel_2, 'push');            app.RunTrainingImagesButton.ButtonPushedFcn = createCallbackFcn(app, @RunTrainingImagesButtonPushed, true);            app.RunTrainingImagesButton.BackgroundColor = [0.9412 0.9412 0.9412];            app.RunTrainingImagesButton.Position = [30 93 147 22];            app.RunTrainingImagesButton.Text = 'Run Training Images';            % Create RunQuantificationButton            app.RunQuantificationButton = uibutton(app.Panel_2, 'push');            app.RunQuantificationButton.ButtonPushedFcn = createCallbackFcn(app, @RunQuantificationButtonPushed, true);            app.RunQuantificationButton.Position = [30 10 147 22];            app.RunQuantificationButton.Text = 'Run Quantification';            % Create LoadTrainingDataButton            app.LoadTrainingDataButton = uibutton(app.Panel_2, 'push');            app.LoadTrainingDataButton.ButtonPushedFcn = createCallbackFcn(app, @LoadTrainingDataButtonPushed, true);            app.LoadTrainingDataButton.Tooltip = {'Select a folder containing ''RefColorMap.mat'' and ''PosColorList.mat'' (optional: ''BackgroundColors.mat'' if checked). Only one of each file name should be in the folder.'};            app.LoadTrainingDataButton.Position = [30 55 147 22];            app.LoadTrainingDataButton.Text = 'Load Training Data';            % Create ORLabel            app.ORLabel = uilabel(app.Panel_2);            app.ORLabel.HorizontalAlignment = 'center';            app.ORLabel.FontWeight = 'bold';            app.ORLabel.Position = [84 74 40 22];            app.ORLabel.Text = '- OR -';            % Create Label_2            app.Label_2 = uilabel(app.Panel_2);            app.Label_2.HorizontalAlignment = 'center';            app.Label_2.FontSize = 14;            app.Label_2.FontWeight = 'bold';            app.Label_2.Position = [2 96 25 22];            app.Label_2.Text = '(1)';            % Create Label_3            app.Label_3 = uilabel(app.Panel_2);            app.Label_3.HorizontalAlignment = 'center';            app.Label_3.FontSize = 14;            app.Label_3.FontWeight = 'bold';            app.Label_3.Position = [2 12 25 22];            app.Label_3.Text = '(2)';            % Create RunProgramLabel            app.RunProgramLabel = uilabel(app.Panel_2);            app.RunProgramLabel.BackgroundColor = [0.8706 0.7059 0.3569];            app.RunProgramLabel.FontSize = 18;            app.RunProgramLabel.FontWeight = 'bold';            app.RunProgramLabel.Position = [0 124 188 26];            app.RunProgramLabel.Text = ' Run Program';            % Create EditField_3            app.EditField_3 = uieditfield(app.Panel_2, 'text');            app.EditField_3.Editable = 'off';            app.EditField_3.Visible = 'off';            app.EditField_3.Position = [30 34 147 22];            app.EditField_3.Value = 'not_yet_chosen';            % Create Panel_3            app.Panel_3 = uipanel(app.UIFigure);            app.Panel_3.AutoResizeChildren = 'off';            app.Panel_3.BackgroundColor = [0.8667 1 0.6902];            app.Panel_3.Position = [13 611 1055 187];            % Create SelectImageFolderButton            app.SelectImageFolderButton = uibutton(app.Panel_3, 'push');            app.SelectImageFolderButton.ButtonPushedFcn = createCallbackFcn(app, @SelectImageFolderButtonPushed, true);            app.SelectImageFolderButton.Position = [7 132 190 22];            app.SelectImageFolderButton.Text = 'Select Image Folder';            % Create EditField            app.EditField = uieditfield(app.Panel_3, 'text');            app.EditField.ValueChangedFcn = createCallbackFcn(app, @EditFieldValueChanged, true);            app.EditField.Editable = 'off';            app.EditField.FontColor = [0.502 0.502 0.502];            app.EditField.Position = [211 132 705 22];            app.EditField.Value = 'please click ''Select Image Folder''';            % Create EnterFileNameFilteroptionalEditFieldLabel            app.EnterFileNameFilteroptionalEditFieldLabel = uilabel(app.Panel_3);            app.EnterFileNameFilteroptionalEditFieldLabel.HorizontalAlignment = 'right';            app.EnterFileNameFilteroptionalEditFieldLabel.Position = [7 103 190 22];            app.EnterFileNameFilteroptionalEditFieldLabel.Text = 'Enter File Name Filter (optional):';            % Create EnterFileNameFilteroptionalEditField            app.EnterFileNameFilteroptionalEditField = uieditfield(app.Panel_3, 'text');            app.EnterFileNameFilteroptionalEditField.ValueChangedFcn = createCallbackFcn(app, @EnterFileNameFilteroptionalEditFieldValueChanged, true);            app.EnterFileNameFilteroptionalEditField.Position = [211 102 100 22];            app.EnterFileNameFilteroptionalEditField.Value = '*.TIF';            % Create canfillinforanynumberofcharactersLabel            app.canfillinforanynumberofcharactersLabel = uilabel(app.Panel_3);            app.canfillinforanynumberofcharactersLabel.Position = [319 91 661 42];            app.canfillinforanynumberofcharactersLabel.Text = {'Use this field to identify the image file type. If desired, select only files with specified characters in the file name'; 'Use * to fill in for any number of characters that are not relevent for file sorting'};            % Create SelectResultDestinationFolderButton            app.SelectResultDestinationFolderButton = uibutton(app.Panel_3, 'push');            app.SelectResultDestinationFolderButton.ButtonPushedFcn = createCallbackFcn(app, @SelectResultDestinationFolderButtonPushed, true);            app.SelectResultDestinationFolderButton.Position = [7 69 190 23];            app.SelectResultDestinationFolderButton.Text = 'Select Result Destination Folder';            % Create EditField_2            app.EditField_2 = uieditfield(app.Panel_3, 'text');            app.EditField_2.Editable = 'off';            app.EditField_2.FontColor = [0.502 0.502 0.502];            app.EditField_2.Position = [211 69 705 22];            app.EditField_2.Value = 'please click ''Select Result Destination Folder''';            % Create EnterNumberofTrainingImagesLabel            app.EnterNumberofTrainingImagesLabel = uilabel(app.Panel_3);            app.EnterNumberofTrainingImagesLabel.HorizontalAlignment = 'right';            app.EnterNumberofTrainingImagesLabel.Position = [6 32 191 23];            app.EnterNumberofTrainingImagesLabel.Text = 'Enter Number of Training Images:';            % Create EnterNumberofTrainingImagesEditField            app.EnterNumberofTrainingImagesEditField = uieditfield(app.Panel_3, 'numeric');            app.EnterNumberofTrainingImagesEditField.LowerLimitInclusive = 'off';            app.EnterNumberofTrainingImagesEditField.Limits = [0 Inf];            app.EnterNumberofTrainingImagesEditField.ValueDisplayFormat = '%.0f';            app.EnterNumberofTrainingImagesEditField.Position = [211 32 35 22];            app.EnterNumberofTrainingImagesEditField.Value = 3;            % Create Label            app.Label = uilabel(app.Panel_3);            app.Label.Position = [250 31 778 23];            app.Label.Text = 'Enter number greater than zero, up to the total number of images in the image folder. Note: training images must all be the same dimensions.';            % Create SubtractBackgroundAreaCheckBox            app.SubtractBackgroundAreaCheckBox = uicheckbox(app.Panel_3);            app.SubtractBackgroundAreaCheckBox.ValueChangedFcn = createCallbackFcn(app, @SubtractBackgroundAreaCheckBoxValueChanged, true);            app.SubtractBackgroundAreaCheckBox.Tooltip = {'If some or all images contain edges of tissue that should not be included in the area calculation'};            app.SubtractBackgroundAreaCheckBox.Text = 'Check here to identify and exclude background area from quantification';            app.SubtractBackgroundAreaCheckBox.Position = [217 3 406 22];            % Create SettingsLabel            app.SettingsLabel = uilabel(app.Panel_3);            app.SettingsLabel.BackgroundColor = [0.6627 0.8196 0.4588];            app.SettingsLabel.FontSize = 18;            app.SettingsLabel.FontWeight = 'bold';            app.SettingsLabel.Position = [1 162 1054 26];            app.SettingsLabel.Text = ' Settings';            % Show the figure after all components are created            app.UIFigure.Visible = 'on';        end    end    % App creation and deletion    methods (Access = public)        % Construct app        function app = DigiPath            % Create UIFigure and components            createComponents(app)            % Register the app with App Designer            registerApp(app, app.UIFigure)            if nargout == 0                clear app            end        end        % Code that executes before app deletion        function delete(app)            % Delete UIFigure when app is deleted            delete(app.UIFigure)        end    endend
