# Supplementary material for: A digital pathology tool for quantification of color features in histologic specimens
Source: Bioeng Transl Med. 2021 Aug 24;7(1):e10242. doi: 10.1002/btm2.10242 (PMC8780932; doi:10.1002/btm2.10242)
Supplement: Supplementary file 3 — Supplemental Figure 1: Hand tracing overlines areas of interest compared to DigiPath. Overlays of hand‐traced (blue) or DigiPath (green) mask on original H&E obstruction (red). Orange box defines area of interest. Supplemental Figure 2: Analysis time with DigiPath and hand tracing. (A) Estimated extrapolation of analysis time for hand tracing (black line) or DigiPath (red line) in larger imaging sets of up to 500 images. Supplemental Figure 3: DigiPath app user interface. The app interface is organized into three sections. The “Settings” section is used to select image folders and file types, assign the number of training images, and choose whether to exclude background areas. The “Run Program” section is used to initiate the training portion of the workflow or load previously generated training data, and to run the automated quantification once the training portion is complete. The “Advanced Settings” section (toggled to visible/invisible) is a panel with additional options to customize aspects of the analysis such as size thresholds, mask color, and morphological operations. Supplemental Figure 4: Kinetics of cell death during cold storage. Quantification of TUNEL staining in Left (top) and Right (bottom) kidneys during cold storage. The ratio of TUNEL‐positive (brown) cell area to TUNEL‐negative (blue) cell area is plotted. Each dot represents one field of view within the biopsy. Lines represent the median. **p < 0.01; ***p < 0.001; ****p < 0.0001. [file BTM2-7-e10242-s003.pdf]

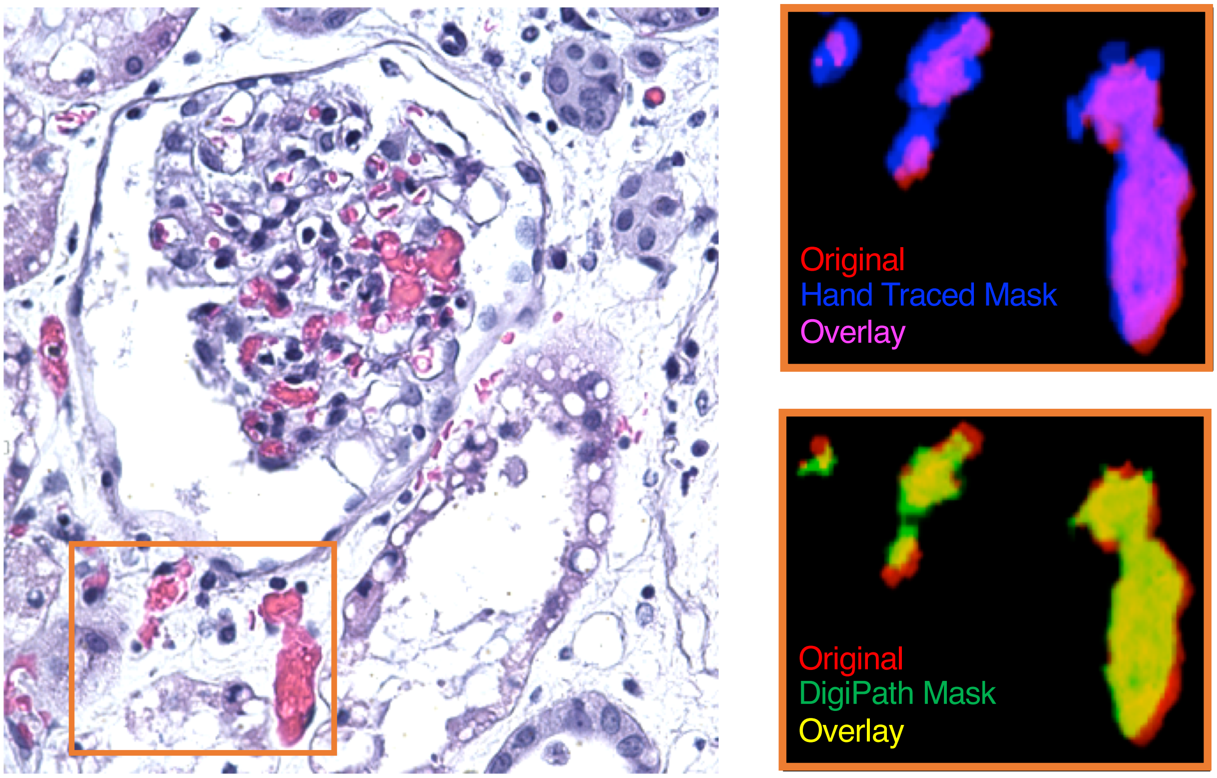

Supplemental Figure 1. Hand tracing overlines areas of interest compared to DigiPath. Overlays of hand-traced (blue) or DigiPath (green) mask on original H&E obstruction (red). Orange box defines area of interest.

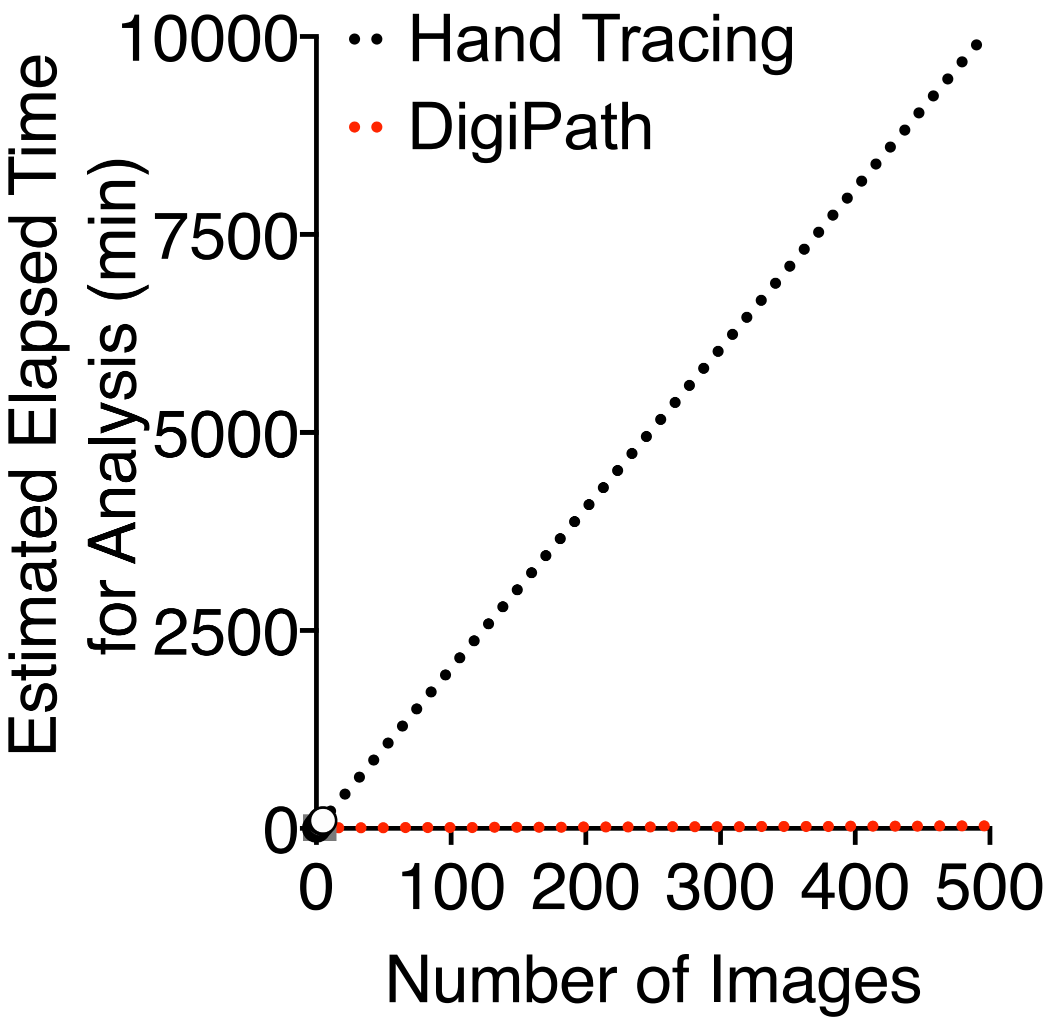

Supplemental Figure 2. Analysis time with DigiPath and Hand Tracing. Estimated extrapolation of analysis time for hand tracing (black line) or DigiPath (red line) in larger imaging sets of up to 500 images.

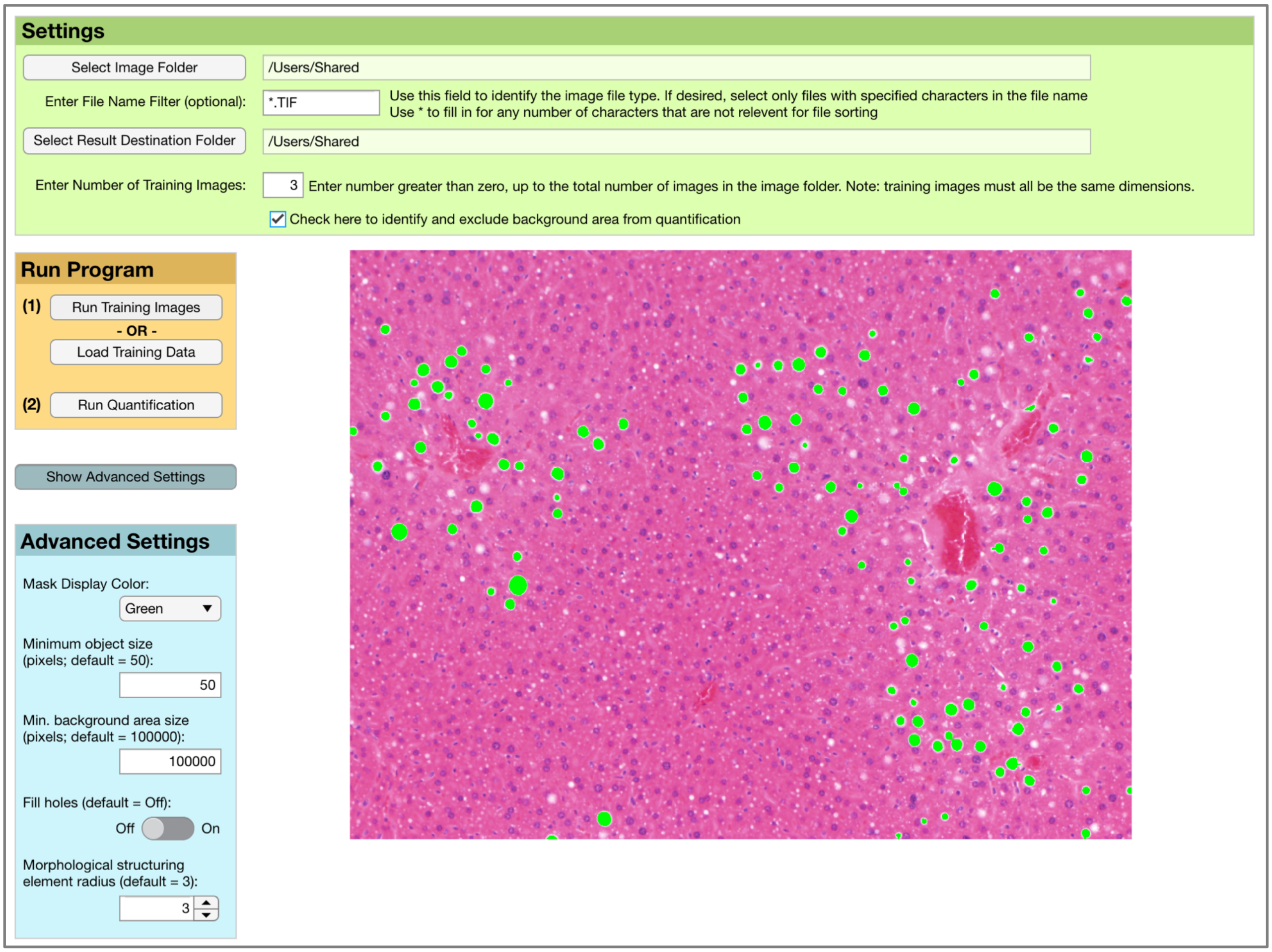

Supplemental Figure 3. DigiPath App User Interface. The app interfaced is organized into three sections. The 'Settings' section is used to select image folders and file types, assign the number of training images, and choose whether to exclude background areas. The 'Run Program' section is used to initiate the training portion of the workflow or load previously generated training data, and to run the automated quantification once the training portion is complete. The 'Avanced Settings' section (toggled to visible/invisible) is a panel with additional options to customize aspects of the analysis such as size thresholds, mask color, and morphological operations.

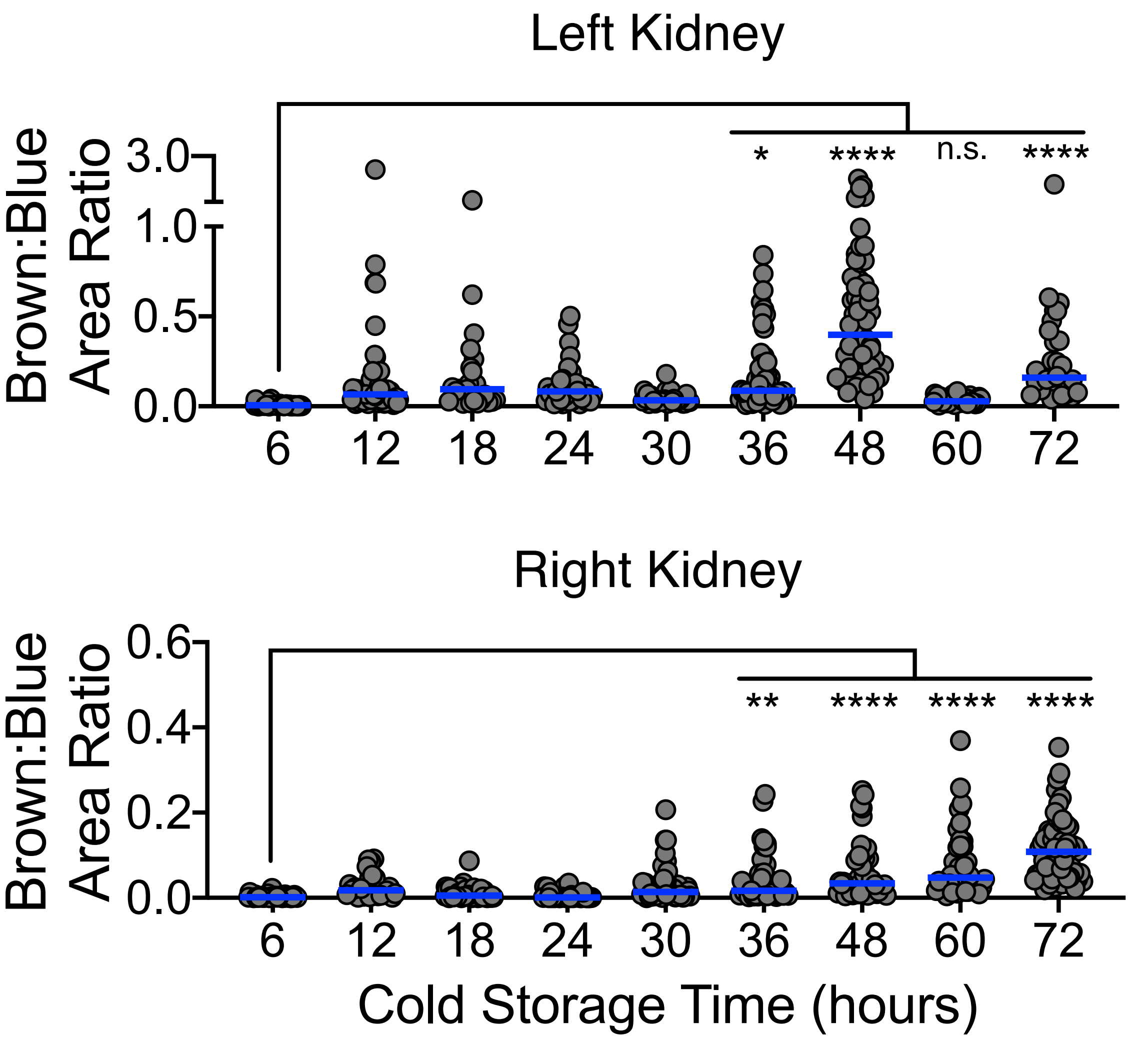

Supplemental Figure 4. Kinetics of cell death during cold storage. Quantification of TUNEL staining in Left (top) and Right (bottom) kidneys during cold storage. The ratio of TUNEL-positive (brown) cell area to TUNEL-negative (blue) cell area is plotted. Each dot represents one field of view within the biopsy. Lines represent the median. **p<0.01; ***p<0.001; ****p<0.0001.
